# Toll-Like Receptor 2 Signaling and Current Approaches for Therapeutic Modulation in Synucleinopathies

**DOI:** 10.3389/fphar.2018.00417

**Published:** 2018-05-04

**Authors:** Ian F. Caplan, Kathleen A. Maguire-Zeiss

**Affiliations:** ^1^Biology Department, Georgetown University, Washington, DC, United States; ^2^Department of Neuroscience, Georgetown University Medical Center, Washington, DC, United States

**Keywords:** TLR, toll-like receptor 2, synucleinopathies, Parkinson's disease, nfkb pathway, inflammation, neuroinflammation

## Abstract

The innate immune response in the central nervous system (CNS) is implicated as both beneficial and detrimental to health. Integral to this process are microglia, the resident immune cells of the CNS. Microglia express a wide variety of pattern-recognition receptors, such as Toll-like receptors, that detect changes in the neural environment. The activation of microglia and the subsequent proinflammatory response has become increasingly relevant to synucleinopathies, including Parkinson's disease the second most prevalent neurodegenerative disease. Within these diseases there is evidence of the accumulation of endogenous α-synuclein that stimulates an inflammatory response from microglia via the Toll-like receptors. There have been recent developments in both new and old pharmacological agents designed to target microglia and curtail the inflammatory environment. This review will aim to delineate the process of microglia-mediated inflammation and new therapeutic avenues to manage the response.

## Introduction

Parkinson's disease (PD), dementia with Lewy bodies (DLB), and multiple system atrophy (MSA) are a group of neurodegenerative diseases called synucleinopathies and are characterized by the presence of Lewy bodies, neuroinflammation, and neuronal loss (Ouchi et al., 2009; Chung et al., 2010). The exact etiology of disease pathogenesis is still debated, yet PD and DLB comprise the second most prevalent neurological disease and second leading cause of dementia, respectively (McKeith et al., 1996; de Lau and Breteler, 2006; Braak and Del Tredici, 2017). The primary constituent of Lewy bodies is α-synuclein (αSyn), from which the name synucleinopathy is given (Spillantini et al., 1997, 1998; Baba et al., 1998; Braak et al., 2003). αSyn garnered significant interest after a number of mutations, namely A53T and A30P, were found in familial cases of PD (Polymeropoulos et al., 1996, 1997; Krüger et al., 1998; Athanassiadou et al., 1999; Singleton et al., 2003; Emmanouilidou et al., 2011). Despite the existence of these familial forms, 90% of PD cases are sporadic and manifest in a predictable clinical pattern as defined by Braak stages 1–6 (Bernheimer et al., 1973; Braak et al., 2003; Fahn, 2003; Braak and Del Tredici, 2008).

A growing body of research has focused on elucidating and modulating the processes of neuroinflammation that occur in synucleinopathies. Within the central nervous system (CNS), microglia are immune cells responsible for surveying and protecting their local environments. The relationship between neuronal loss and microglial activation is not definitively established, but irrespective of the disease initiating event microglial activation seems to be an integral potentiator of the disease process (as reviewed by; Block et al., 2007). Neurons express and release αSyn into the surrounding environment (Lee et al., 2005; Kim C. et al., 2016) and microglia recognize this with Toll-like receptors (TLRs) and subsequently produce a proinflammatory response (Zhang et al., 2005; West et al., 2006; Su et al., 2008; Vezzani et al., 2011). The importance of microglia-mediated inflammatory responses is strengthened by the observation that dopaminergic neuron loss is correlated with microglial upregulation of inducible nitric oxide synthase (iNOS) and the release of nitric oxide (NO) (Knott et al., 2000; Gao et al., 2008), tumor necrosis factor α (TNFα) (McCoy et al., 2006; Takeuchi et al., 2006), and interleukin (IL)−1β (Ferrari et al., 2006). This inflammatory state can be measured with [^11^C](R)-PK11195 positron emission tomography (PET) imaging and such studies demonstrate progressive microglial activation during early pathogenesis, followed by sustained microglial activation during mid to late disease stages (Gerhard et al., 2006a; Ouchi et al., 2009). Relevant to synucleinopathies, amoeboid microglia, a morphology typical of activation, are associated with disease pathology (αSyn) in the brains of patients with diagnosed Parkinson's disease or incidental Lewy Body disease (Doorn et al., 2014). Furthermore, increased microglial TLR2 expression was found in a subset of these affected brains (Doorn et al., 2014). Additional support for a role of TLR2-mediated inflammation in Parkinson's disease comes from Droiun-Ouellet et al., where TLR2 expression was increased in circulating monocytes of Parkinson's patients (Drouin-Ouellet et al., 2015). Therefore, this review aims to focus on the relationship between the particulars of αSyn and the TLR2 signaling pathway with respect to their roles in synucleinopathy progression and recent advances in inhibiting the microglial inflammatory response.

## α-synuclein and the innate immune response of the CNS

The innate immune system is charged with differentiating between the self and other in order to maintain the health of the periphery and CNS. Pattern recognition receptors (PRRs) are crucial in mediating host defenses to invading pathogens. Pathogen-associated molecular patterns (PAMPs), which include various bacterial and viral components, are the exogenous molecules that PRRs, such as RIG-I-like receptors, NOD-like receptors, and TLRs respond to (as reviewed by; Chen and Nunez, 2010).

### Toll-like receptors

TLRs are named after the original gene that was identified in *Drosophila* and they are integral to the innate immune response. Thus far 13 mammalian TLRs have been identified, with humans expressing TLRs 1–10 and mice expressing TLR1-9 and 11–13. All 13 members are single pass transmembrane proteins with the C terminal located intracellularly and the N-terminal, which contains the distinctive leucine-rich repeats, situated extracellularly and acting as the ligand recognition domain (Matsushima et al., 2007). TLRs 1, 2, 4, 5, 6, and 10 are located on the plasma membrane and recognize PAMPs from the extracellular space. In contrast, TLRs 3, 7, 8, 9, 11, 12, and 13 are located on intracellular endosomes and are responsible for recognition of internalized PAMPs including both bacterial and parasitic DNA as well as viral single- and double-stranded RNA (as reviewed by; Akira et al., 2006; Kawai and Akira, 2007).

**Figure 1:**
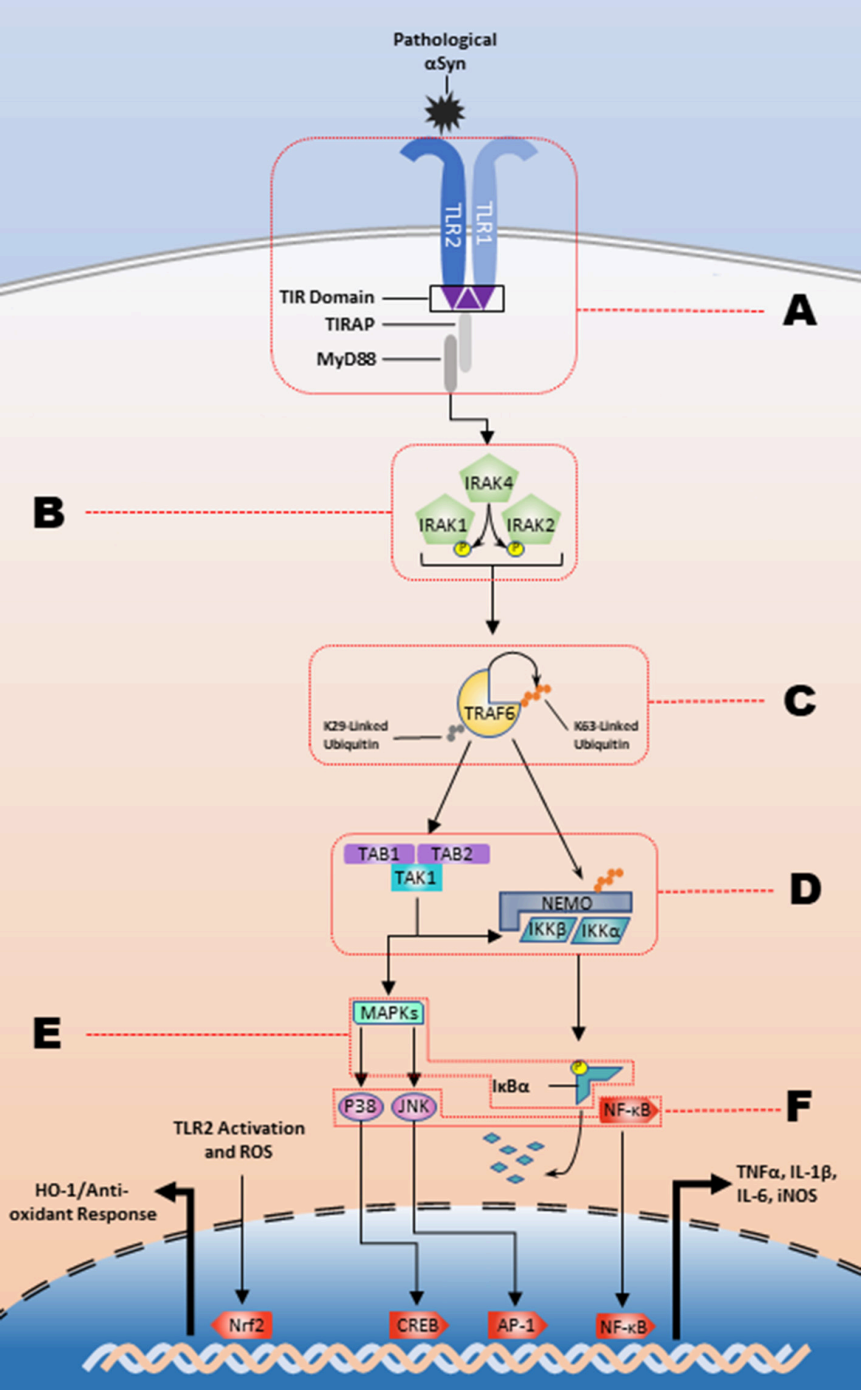
The TLR2/1 signaling cascade and respective regulatory nodes. αSyn binding to the TLR2/1 heterodimer leads to a MyD88-dependent response that stimulates the kinase activity of the IRAK complex. The IRAK complex in turn activates TRAF6 K63-linked auto-ubiquitination, which subsequently leads to the release of the IKKs and activation of TAK1. The IKKs will designate IκBα for degradation and TAK1 will stimulate the MAPK pathway leading to the NF-κB, JNK, and p38 nuclear translocation to upregulate proinflammatory cytokines. There are several potential regulatory nodes and letters A-F represent those targets for intervention along the signaling pathway in order to impede pathogenesis.

The inflammatory response is contingent upon the intracellular interactions of these signaling pathways. Intracellular C-terminal domains contain Toll-interleukin 1 receptor (TIR) domains, which are responsible for transforming extracellular recognition to an intracellular response (Xu et al., 2000; Horng et al., 2002; Brown et al., 2006). To date, 5 adaptor molecules have been identified that facilitate TLR signaling and lead to the differential cellular responses to varying stimuli: MyD88, TRIF, TRAM, TIRAP/Mal, and Sarm1. TRAM and TIRAP function to recruit MyD88 and TRIF to their respective TLRs and all TLRs, except for TLR3, activate the MyD88-dependent pathway (as reviewed by Kawai) (Horng et al., 2002; Kawai and Akira, 2010). The TRIF-dependent pathway signals through downstream kinases, TANK binding kinase 1 (TBK1) and IKKε, to activate IRF3 and subsequently produce type 1 interferons (Yamamoto et al., 2002b; Oshiumi et al., 2003). In the MyD88-dependent pathway (Figure 1), death domain interactions mediate intracellular signal transduction in a sequential manner from MyD88 to the phosphorylation of interleukin-1 receptor-associated kinase (IRAK) 4, then to IRAK1 and IRAK2 (Lin et al., 2010). The IRAK complex interacts with TNF receptor associated factor 6 (TRAF6) which will undergo K63-linked autoubiquitination and will ubiquitinate NF-κB essential modulator (NEMO). This is followed by the activation of the complex of transforming growth factor-β-activated kinase-1 (TAK1), TAK1 binding protein (TAB)2, and TAB3. TAK1 subsequently phosphorylates IKKα and IKKβ, and the IKKs will phosphorylate IκBα marking it for degradation. This ultimately results in production of proinflammatory cytokines through NF-κB, the heterodimeric p50/p65 protein, nuclear translocation and MAPK activation (as reviewed by) (Johnson and Lapadat, 2002; Symons et al., 2006; Kawai and Akira, 2007). As an example, the prototypical stimulator of this pathway, the TLR4 agonist bacterial-derived lipopolysaccharide (LPS) (Poltorak et al., 1998), causes an increase in the production of iNOS (Kacimi et al., 2011), and proinflammatory cytokines such as IL−1β, IL-6, and TNFα (Yamamoto et al., 2002a). However, several studies suggest that endogenous ligands, such as heparan sulfate, heat shock proteins, and high-mobility group box 1 (HMGB1) can stimulate TLR signaling suggesting a role for sterile inflammation in diseases like synucleinopathies, which have the hallmark feature of increased amounts of misfolded endogenous proteins (Yu et al., 2010).

### α-synuclein: structure and function

αSyn is a pathogenic protein, which accumulates in all synucleinopathies and is hypothesized to propagate sterile inflammation in these disorders. TLRs, while recognizing foreign PAMPs, are also reactive to damage/danger-associated molecular patterns (DAMPs). DAMPs, such as αSyn, are endogenous molecules that are intracellularly innocuous, but upon secretion or release following cellular injury will stimulate an innate immune response.

The primary function of αSyn under normal conditions has not been definitively described. However, αSyn is localized to axon terminals where it interacts with more than 50 other proteins and plays a role in vesicular transport. The protein is encoded by the gene *SNCA* that is located on chromosome 4 and evidence from *SNCA* knockout mice shows the loss of αSyn leads to hindered docked vesicle replenishment, which indicates a possible role for αSyn in vesicular trafficking between the reserve and readily releasable pools of neurotransmitter vesicles. Other potential *in vivo* functions for αSyn include enzymatic regulation, transporter regulation, mitochondrial function, and cell survival. Much support for the pathogenic role of αSyn comes from genetic mutations within *SNCA* that lead to familial cases of PD and evidence showing that triplication of wildtype *SNCA* leads to increased risk for development of PD. These data support a framework where both mutated αSyn and an overexpression of αSyn can be pathogenic (Singleton et al., 2003). Furthermore, evidence of αSyn-mediated inhibition of tyrosine hydroxylase (TH) and upregulation of dopamine transporters strengthens the case for the importance of αSyn in PD (as reviewed by; Dev et al., 2003; Uversky, 2007; Breydo et al., 2012; Lashuel et al., 2012; Deleersnijder et al., 2013).

αSyn is a 140 amino acid protein with a primary structure that can be divided into three domains. α-Helices compose the first 60 amino acids, the following 35 amino acids are largely hydrophobic, and the final 45 amino acids are acidic (Uversky, 2011b). The efforts to characterize the native conformation of αSyn have led to two proposals; one provides support for a stable tetramer formation that is ostensibly lost after cell lysis (Dettmer et al., 2013) and the other body of evidence shows αSyn to be an intrinsically disordered protein (IDP) with an ill-defined secondary structure and a very compact tertiary structure (as reviewed by) (Uversky, 2011a; Breydo et al., 2012; Deleersnijder et al., 2013). Both of the above hypotheses are focused on the monomeric or low-molecular weight structure of αSyn, but it is not these species that seems to be driving the activation of TLRs.

### Progression of misfolding and production of neurotoxic species

As mentioned above, αSyn is a presynaptic protein and thus Lewy body pathology is most often found in intraneuronal inclusions, except in multiple system atrophy where αSyn pathology is localized to oligodendrocytes. The ability for αSyn to have deleterious inflammatory effects on microglia originates from neuronal exocytosis, which was demonstrated in an *in vitro* αSyn overexpression model using SH-SY5Y neuroblastoma cells. Normal SH-SY5Y cell media has no effect on microglial proinflammatory production, but SH-SY5Y cells overproducing wildtype or A53T mutant αSyn induce the microglial proinflammatory response (Lee et al., 2010; Alvarez-Erviti et al., 2011). In particular relevance to PD, greater protofibril αSyn stabilization occurred in the presence of dopamine (Conway et al., 2001). Furthermore, results from Xu et al. illustrate that αSyn-mediated neurotoxicity of substantia nigra dopamine neurons is due, in part, to αSyn interactions with dopamine synthesis that creates reactive oxygen species (ROS) leading to neuronal death (Xu et al., 2002). While this corroborates a membrane-permeabilization/apoptotic hypothesis of disease pathogenesis, there is also evidence to suggest that neurons spontaneously exocytose vesicles rich in αSyn (Lee et al., 2005). This ER/Golgi-independent exocytotic mechanism is exacerbated under cellular stress conditions, such as proteasome inhibition (Jang et al., 2010). The extracellular αSyn can then be endocytosed by neighboring neurons and this neuron-to-neuron transmission has been shown to be neurotoxic (Desplats et al., 2009).

The process of αSyn aggregation and propagation is not conclusively understood. For example, the creation of high order oligomers can be initiated through a number of changes including the presence of metal ions, oxidative stress (Hashimoto et al., 1999), molecular crowding (Shtilerman et al., 2002), and environmental factors such as pesticides (Lai et al., 2002). During fibrillization the IDP nature of monomeric αSyn is converted to a β-sheet structure, which forms amyloid fibrils that are comparable to Aβ in Alzheimer's disease. These αSyn fibrils are composed of multiple proteins that stack in anti-parallel β-sheets, eventually becoming a primary constituent of Lewy bodies (Conway et al., 1998, 2000).

The intermediate steps that bridge the folding of a monomer to an amyloid fibril are potentially the most critical when considering the driving force behind synucleinopathies. Fibrillization occurs through the formation of partially folded intermediate structures that exist as membrane localized homodimers and annular structures (Ding et al., 2002; Tsigelny et al., 2008). The presence of polyunsaturated fatty acids, which are the major component of lipid membranes, enhance oligomer formation and have been found to be elevated in PD and DLB brains, compared to age-matched healthy controls (Perrin et al., 2001; Sharon et al., 2003). There is also evidence to suggest that pathological phosphorylation at Ser-129 of αSyn potentiates the fibrilization process (Fujiwara et al., 2002). Interestingly, evidence suggests that it is these oligomeric structures, and not the fibrils, that are neurotoxic due to an increase in membrane permeabilization (Volles et al., 2001; Volles and Lansbury, 2002). Karpinar et al. generated a number of αSyn variants with proline residue substitutions in three different locations of the primary amino acid sequence. The mutants were much less likely to form highly organized fibrils and were more toxic than wildtype αSyn samples due to the increase in protofibril structures. Furthermore, in *Drosophila*, these mutations led to a reduction of TH^+^ cells in the lateral and medial brain fractions (Karpinar et al., 2009). Multiple reports show that it is high molecular weight αSyn and not monomeric conformer, that leads to microglia activation and TNFα release (Su et al., 2008; Lee et al., 2010; Béraud et al., 2013; Fellner et al., 2013; Daniele et al., 2015). However, contradictory evidence from a THP-1 cell model demonstrates fibrils, and not monomers or oligomers of αSyn, are able to generate an inflammatory response (Gustot et al., 2015). It should be noted that αSyn treatment conditions vary between laboratories and may be the reason for disparate experimental outcomes. Furthermore, the folding mechanisms are difficult to assess *in vivo* (Danzer et al., 2007).

## Microglial and neuronal expression of TLR2/1

TLR2 is a member of the MyD88-dependent receptor family that is associated with a diverse set of ligands. TLR2 interacts with peptidoglycan, Gram-positive bacteria, lipoproteins, and the yeast cell wall component zymosan (Underhill et al., 1999a,b; Ozinsky et al., 2000; Takeuchi et al., 2000, 2002; Morr et al., 2002). In order to associate with and recognize these various agonists TLR2 requires a coreceptor, typically TLR1 or TLR6. The TLR2/1 heterodimer is activated by Pam_3_CSK_4_, a synthetic tri-acylated lipopeptide, highlighting the specificity for tri-acylated lipoproteins (Ozinsky et al., 2000; Takeuchi et al., 2001, 2002; Buwitt-Beckmann et al., 2006). TLR2 activity is also implicated in non-immune functions, such as the potential to regulate metabolism and protect against age-related obesity (Shechter et al., 2013).

Within the cell, TLR2 and TLR1 colocalize with one another both intracellularly and on the plasma membrane, demonstrating that the two receptors are potentially in their heterodimeric state prior to any ligand interaction. Sandor et al. used chimeric TLR2 and TLR1 proteins to investigate each receptor's role in NF-κB translocation and found both intracellular TIR domains were needed to recruit MyD88 and propagate a signal (Sandor et al., 2003). Upon binding of Pam_3_CSK_4_, TLR2/1 adopts an “m” shaped conformation with the two C-terminal domains interacting in the interior intracellular region and each N-terminal arching out extracellularly. The Pam_3_CSK_4_ binding domain is found at the pocket formed between the central and C-terminal domains, a convex region which is atypical for the leucine rich receptor family (Jin et al., 2007). It is postulated that there are hydrophobic portions of TLR2/1 that could be involved in large DAMP recognition (Nishiguchi et al., 2001; Okusawa et al., 2004; Jin et al., 2007). Liang et al. demonstrated that the pentameric B subunit of type IIb (LT-IIb-B), a doughnut shaped oligomeric protein from *E. coli*, had 10 potential binding sites on TLR2/1. The convex region of TLR2 is postulated as the most likely region where LT-IIb-B binds (Liang et al., 2009).

A growing body of evidence establishes a role for TLRs expressed on microglia to mediate cell death in the neural environment. For example, stimulation of TLR4 leads to a loss of oligodendrocytes and axons that is not seen when microglia are absent (Lehnardt et al., 2003). TLRs also play an important role in pain (as reviewed by; Nicotra et al., 2012). Tanga et al. demonstrated that TLR4 contributes to early neuropathic pain associated with nerve transection suggesting that while these receptors are conserved mediators of pathogen recognition and removal, TLRs also have the potential to accelerate neurodegeneration upon activation (Tanga et al., 2005). Similarly, TLR2 (Jana et al., 2008), TLR4, and CD14 are implicated in Alzheimer's disease as integral receptors for fibrillar Aβ recognition (Reed-Geaghan et al., 2009). Within the field of synucleinopathy research, oligomeric forms of αSyn have been shown to directly interact with the TLR2/1 heterodimer and lead to a prototypical inflammatory response (Kim et al., 2013, 2015; Daniele et al., 2015). Other reports support a role for TLR4 in αSyn recognition, where this ligand-receptor interaction facilitates a phagocytic response (Stefanova et al., 2011). Taken together, the above investigations support that αSyn binds to TLR MyD88 family members and in the case of TLR2/1 this interaction may occur in a similar fashion as LT-IIb-B, resulting in activation of this heterodimeric receptor on microglia and contributing to pathogenesis. These studies provide rationale supporting the hypothesis that the oligomeric, and not monomeric, species of αSyn are responsible for disease pathology.

### In microglia

Microglia reside in the CNS and function as the resident immune cells of their local environments. Morphologically, inactive microglia are filamentous, continually extending and retracting processes surveying their surroundings (Davalos et al., 2005; Nimmerjahn et al., 2005). Microglia possess the capacity for recognition of various pathogens and endogenous cues, which produce further morphological changes that are typically described as the M1 or M2 phenotypes (de Sousa et al., 2011; Wei et al., 2011). This binary categorization is meant to represent the prototypical proinflammatory and anti-inflammatory/phagocytic responses of microglia (Bachstetter et al., 2014; Majerova et al., 2014). But more recent investigation has begun to reorganize this nomenclature in favor of a continuum of morphological characteristics, each of which being representative of a particular microglial function (as reviewed by; Hanisch, 2013; Kim C. C. et al., 2016; Morganti et al., 2016; Ransohoff, 2016). The heterogeneity of phenotypes is further evidenced by age and region specific analyses that show the great diversity of microglia (Grabert et al., 2016).

TLRs are integral to microglia, and the microglial expression of the TLR2/1 heterodimer is specifically implicated in synucleinopathy pathogenesis. αSyn or Pam_3_CSK_4_ activation of TLR2 in BV-2 microglia (Béraud et al., 2013), primary mouse microglia (Babcock et al., 2006; Downer et al., 2013; Kim et al., 2013; Daniele et al., 2015), and human microglia leads to the MyD88-dependent release of TNF-α and IL-1β (Dzamko et al., 2016). Following stimulation of TLR2/1, TLR2 mRNA and protein are increased in microglia (Syed et al., 2007). Studies such as this provide rationale for a cyclical process of inflammation, where once an initial insult is recognized the microglia are primed for sustained proinflammatory responses. Further investigation shows that αSyn overexpressing SH-SY5Y cells release oligomeric αSyn, the SH-SY5Y conditioned media is capable of activating microglia, and microglia TLR2 expression is required for this activation (Kim et al., 2013; Kim C. et al., 2016). The activation profile of microglia exists somewhere within the spectrum of the prototypical M1 and M2 states when pretreated with non-aggregated αSyn and subsequently activated with Pam_3_CSK_4_ (Roodveldt et al., 2013), supporting the dynamic phenotype model of microglia and suggesting that *in vivo* microglial activation via oligomeric αSyn could result in paracrine/autocrine TNF-α signaling that potentiates microgliosis and neuronal death.

The microglial response via TLR2/1 is not a homogenous proinflammatory response. Rather, 24 h after αSyn stimulation microglia also produce IL-10, an anti-inflammatory cytokine (Daniele et al., 2015). The endogenous Ras-ERK inhibitors, downstream of kinase (Dok) 1 and 2, are also upregulated in microglia following TLR2/1 stimulation. While ERK phosphorylation is also increased after TLR2 activation, the upregulation of Dok1 and Dok2 represent another inhibitory signal that is upregulated to limit the immediate inflammatory response of microglia (Downer et al., 2013). There is also an antagonistic pathway mediated by the nuclear factor erythroid 2-related factor 2 (Nrf2)/antioxidant response element (ARE) pathway, which transcriptionally upregulates heme oxygenase-1 (HO-1) in the CNS as well as the periphery (Lee and Suk, 2007; Koh et al., 2011). The proinflammatory response from microglia generates reactive oxygen species (ROS), which in turn stimulates the Nrf2/ARE pathway as a late phase response to control microglial activation (Li et al., 2008; Innamorato et al., 2009). The Nrf2/ARE and the NF-κB pathways cross regulate one another, which provides a unique target for managing microglial responses to αSyn and proinflammatory cytokines (Innamorato et al., 2008; Liu et al., 2008).

### In neurons

Neurons themselves are also capable of expressing TLRs. Evidence suggests that TLRs play a role in neuronal morphogenesis, neurodegeneration, and cytokine production (as reviewed by) (Liu H. Y. et al., 2014). TLR2 is expressed in NT2-N teratocarcinoma cells (Lafon et al., 2006), SH-SY5Y neuroblastoma cell (Kim et al., 2015), primary mouse neurons (Downer et al., 2013), and human neurons in the CNS (Dzamko et al., 2016) and ENS (Brun et al., 2013). Very interestingly, Dzamko et al. surveyed 17 human PD brains and found a statistically significantly age-related increased in neuronal TLR2 expression (Dzamko et al., 2016). One group showed that neuronal transmission of αSyn can be mediated by lymphocyte-activation gene 3 (LAG3), which binds to αSyn fibrils during neuronal endocytosis (Mao et al., 2016). Both SH-SY5Y and induced pluripotent stem cell-derived neurons amass αSyn after Pam_3_CSK_4_ but not LPS treatment, suggesting neuronal TLR2/1 stimulation upregulates αSyn expression and/or accumulation in neurons (Dzamko et al., 2016). Together these studies suggest that αSyn can act in a paracrine/autocrine fashion to activate both microglial and neuronal TLR2/1 in order to potentiate microglial activation and neuronal degeneration.

While neurons can express and release proinflammatory cytokines, some evidence suggests that the primary effect of αSyn-mediated TLR2/1 signaling is through an inhibition of the autophagy pathway. Kim et al. demonstrate TLR2 signaling increases mTOR and Akt phosphorylation, which impairs the autophagy (Kim et al., 2015). The Akt phosphorylation may be mediated by TRAF6 K63-linked ubiquitination (Yang et al., 2009). When assessing potential causes for neuronal loss, this evidence provides insight into potential mechanisms of αSyn-related neuronal autophagocytotic dysregulation that could contribute to synucleinopathy pathology. In this setting, impaired autophagy would lead to an accumulation and release of neuronal αSyn making this protein available as an endogenous TLR ligand.

## Inhibiting the inflammatory pathway

As the evidence supporting a pathological role for TLR2/1 signaling pathway has increased, there has been a heightened interest in discovering potential avenues to modulate this pathway for therapeutic benefits. The interdisciplinary findings from both neuroscience and immunology have helped provide supporting data and novel ways to treat synucleinopathies. The spectrum of potential interventions to inhibit the various nodes of the pathway is depicted in Figure 1 and includes modulation of endogenous microRNA's and proteins, as well as treatment with both naturally derived, and synthetic compounds, described in Table 1, for the purpose of treating diseases characterized by inflammatory environments.

**Table 1:** Modulators of the TLR2/1 signaling pathway.

**Inhibitor**	**Target**	**References**
**MicroRNAs**		
miR-UL112-3p	TLR2	Vo et al., 2005
miR-K5	IRAK1	Xiao and Rajewsky, 2009
miR-K9	MyD88	Xiao and Rajewsky, 2009
miR-143	TLR2 and NF-κB	Krichevsky et al., 2003; Wayman et al., 2008
miR-132	IRAK4	Abend et al., 2012
miR-21	MyD88 and IRAK1	O'Neill et al., 2011
miR-146a	TRAF6 and IRAK1	Chen et al., 2013a; Guo et al., 2013; Landais et al., 2015; Liu et al., 2015
miR-124	TRAF6	Taganov et al., 2006; Nahid et al., 2013
miR-92a	MKK4	Meisgen et al., 2014
miR-147 miR-7116-5p	Unknown TNFα	Saba et al., 2012; Ma et al., 2014; Santra et al., 2014
miR-155	SOCS1 and SHIP1	Liu et al., 2009; Zhang et al., 2016
**NATURAL COMPOUNDS**		
Methylpenicinoline	Unknown	Strassheim et al., 2005
Calcitriol	miR-155	Nakagawa et al., 2002
Schizandrin A/B	Unknown	Aravalli et al., 2008; Aslanidis et al., 2015; Koymans et al., 2015
Gomosins A/G/J/N Pristimerin	Nrf2 IRAK1 and TRAF6	Chen et al., 2013b; Kim et al., 2014
Deoxysappone B	Unknown	Song et al., 2016
Sparstolonin B	MyD88	Park et al., 2014
Daphnetin	A20	Kumar et al., 2016
Curcurbitacin B/E/I	Nrf2	Liu et al., 2016
Kolaviron	Nrf2	Yao et al., 2011
Pseudoginsenoside-F11	MyD88	Park et al., 2015
**SYNTHETIC COMPOUNDS**		
CU-CPT22	TLR2/1	Arel-Dubeau et al., 2015; Olajide et al., 2017
RSCL-0409	MyD88	Oyagbemi et al., 2017
C_16_H_15_NO_4_ (C29)	MyD88	Wang et al., 2014
*Ortho*-vanillin	MyD88	Wang et al., 2014
M2000	Unknown	Kalluri et al., 2010; Mistry et al., 2015
G2013	Unknown/Tollip	Afraei et al., 2015; Mirshafiey et al., 2016; Aletaha et al., 2017
NG25	TAK1	Pourgholi et al., 2017
MRT67307	TBK1 and IKKε	Jahromi et al., 2017
BMS345541	IKKβ	Nazeri et al., 2017
MCAP	Unknown	Hajivalili et al., 2016
Bay-11-708	NF-κB	Sharifi et al., 2017
Candesartan	Angiotensin II receptor	Pauls et al., 2012
Fasudil	Rho kinase	Yang et al., 2006; Clark et al., 2011
cRGD	MFG-E8 receptor	Matteucci et al., 2015

### microRNA regulation

microRNAs are a class of non-coding RNAs that are approximately 23 nucleotides in length. They act as translational repressors (Baek et al., 2008) and are involved in development (Wightman et al., 1991, 1993; Vo et al., 2005), neurogenesis (Krichevsky et al., 2003), and plasticity (Wayman et al., 2008). These small regulatory microRNA's are also instrumental in the endogenous regulation of the immune system and specifically TLR signaling (as reviewed by; Xiao and Rajewsky, 2009; O'Neill et al., 2011).

The membrane localized site of node A (Figure 1) is a target for multiple microRNAs. The exogenous miR-UL112-3p, a product of the human cytomegalovirus, directly binds the 3' untranslated region of TLR2 (Landais et al., 2015). The endogenous miR-143 downregulates TLR2 in hepatoma cells (Liu et al., 2015) and colorectal carcinoma cells (Guo et al., 2013). The receptor-adaptor complex is also known to be regulated at MyD88 by miR-21 (Chen et al., 2013a) and miR-K9, which is a product of Kaposi's sarcoma-associated herpesvirus (Abend et al., 2012).

A larger collection of microRNAs has been identified that have inhibitory effects on TLR2 signaling (Figure 1; Node B). IRAK1 is targeted by miR-K5 (Abend et al., 2012), again from Kaposi's sarcoma-associated herpesvirus, miR-21 (Chen et al., 2013a) and miR-146a (Taganov et al., 2006; Nahid et al., 2009, 2011; Quinn et al., 2013). Additionally, IRAK4 is inhibited by the TLR2-upregulated and CREB-dependent miR-132 in THP-1 cells, human PBMCs, and murine microglia (Nahid et al., 2013). Further along the signaling pathway (Figure 1; Node C) the auto-ubiquitinating TRAF6 has been intensely investigated with relation to THP-1 cells (Taganov et al., 2006; Nahid et al., 2011; Quinn et al., 2013), keratinocytes (Meisgen et al., 2014), PBMCs (Nahid et al., 2013), oligodendrocytes (Santra et al., 2014), and microglia using TLR2, TLR4, and prion-induced mechanisms (Saba et al., 2012). miR-146a inhibits the translation of TRAF6, while miR-146a knockdown leads to an increased production of proinflammatory cytokines. TRAF6 is also a target of miR-124, which inhibits the inflammatory response (Ma et al., 2014; Qiu et al., 2015). Activation of TLR2, or stimulation via the actin regulator thymosin β4, leads to the subsequent upregulation of miR-146a that lasts up to 4 days post-stimulation, which signifies the robust endogenous response following TLR2 activation that is created to modulate the inflammatory response (Meisgen et al., 2014; Santra et al., 2014). There is also microRNA inhibition at node E (Figure 1) that indicates additional layers of endogenous regulation of the TLR2 activation pathway. Overexpression of miR-92a inhibits TNFα and IL-6 release through inhibition of MKK4, a MAPK family protein, and is downregulated following TLR2 stimulation (Lai et al., 2013). A recent study has also identified miR-7116-5p as a negative regulator of TNFα protein production and the MPTP model of PD leads to a downregulation of miR-7116-5p (He et al., 2017).

It is also important to note that not all TLR-associated microRNAs have known functions or downregulate the pathway. The target of miR-147 is unknown, but miR-147 is upregulated following TLR2 and TLR4 stimulation and reduces proinflammatory cytokine production (Liu et al., 2009). Further investigation has shown miR-147 suppresses Akt expression in breast cancer cell lines (Zhang et al., 2016). These data suggest a possible complimentary mechanism for miR-147 where it may be able to limit the proinflammatory response, but it could also be promoting the autophagy pathway. A prototypical example of TLR2 signaling potentiation is miR-155, which is upregulated with TLR4 activation via both the AP-1 (O'Connell et al., 2007; Yin et al., 2008) and NF-κB pathways (Bala et al., 2011). miR-155 increases the production of TNFα and other cytokines through its inhibition of suppressor of cytokine signaling 1 (SOCS1) (Cardoso et al., 2012) and Src homology 2 domain-containing inositol-5-phosphatase 1 (SHIP1) (Thounaojam et al., 2014).

The manipulation of microRNAs as a therapeutic strategy is a unique and interesting approach to modifying synucleinopathy pathogenesis. Exogenous viral microRNAs are likely used in order for the viruses to invade the host immune system and the properties of miR-UL112-3p, miR-K5, and miR-K9 could be exploited for therapeutic effects. But it is also important to note that Jurkin et al. demonstrated alternative expression profiles of miR-146a in different cell types (Jurkin et al., 2010). This indicates the need for a more complete understanding of how each microRNA functions within the CNS prior to the development of synucleinopathy therapies.

### Endogenous proteins

**Figure 2:**
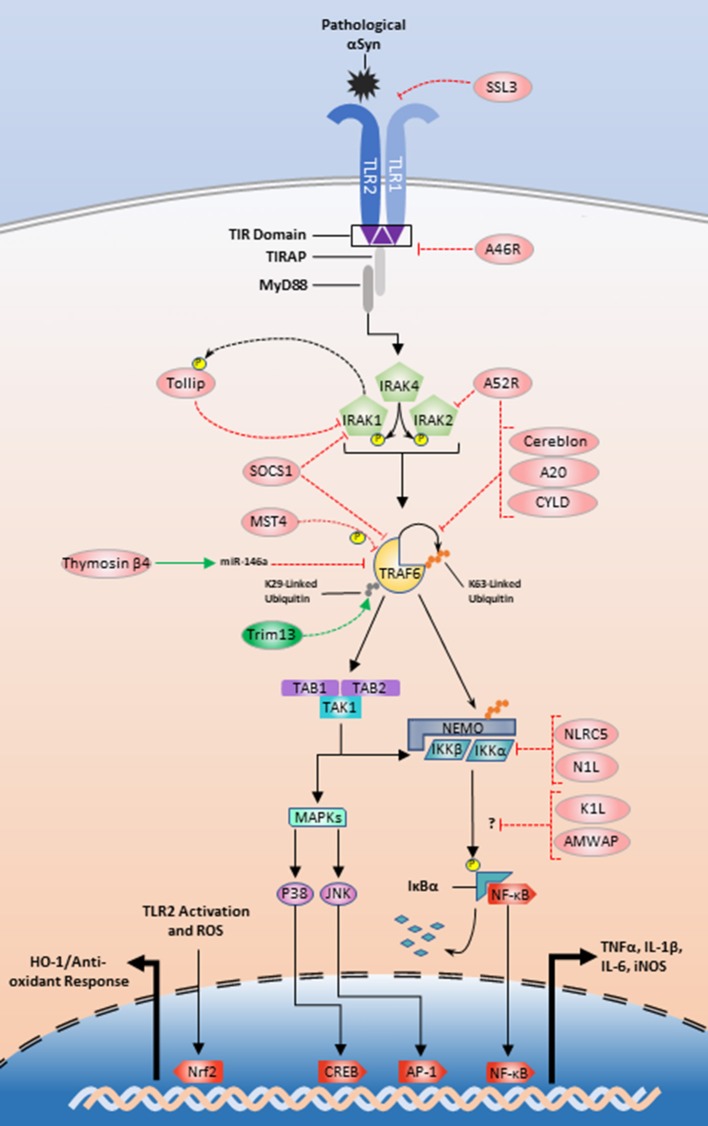
Exogenous and endogenous protein regulation of the TLR2/1 signaling pathway. Initial regulation could begin with SSL3 inhibition of αSyn recognition or A46R disturbance of the MyD88-TIR interface (Node A; Figure 1). Tollip, A52 and SOCS1 could be manipulated to inhibit signaling at the IRAK complex (Node B; Figure 1). TRAF6 inhibition could be achieved by interference with Trim13 or positive modulation of SOCS1, MST4, thymosin β4, cereblon, A52R, A20, and CYLD (Node C; Figure 1). Lastly NLRC5 and N1L could be positively upregulated to inhibit the IKKs (Node D; Figure 1). The modes of action for K1L and AMWAP are not established, but these two proteins along with upregulation of the transcription factor Nrf2 are also potential targets for TLR2/1 modulation.

Continuing efforts to manipulate the TLR2/1 inflammatory pathway also utilize endogenous-protein regulators in conjunction with microRNAs. As mentioned above, thymosin β4 is an example of an endogenous protein that promotes miR-146a upregulation and subsequent TRAF6 and IRAK1 translational repression (Santra et al., 2014; Zhou et al., 2015). IRAK1 is also regulated by Tollip, which is a substrate for IRAK1 phosphorylation that interacts in a negative feedback loop (Figure 2), to inhibit IRAK1 and limit the production of TNFα, IL-1β, and IL-6 (Zhang and Ghosh, 2002; Didierlaurent et al., 2006; Shah et al., 2012). Interestingly, Tollip is highly expressed within the midbrain region of mice, making it a high priority target in relation to PD (Humbert-Claude et al., 2016).

TRAF6 is also an emerging target for TLR modulation and looks to be tightly and multiply regulated (Figure 2). A large structural analysis study identified two de-ubiquitinases, A20 and CYLD, as endogenous regulators of TRAF6 K63-linked ubiquitin chains (Guven-Maiorov et al., 2015). Using a THP-1 model, Jiao et al. showed that MST4 directly phosphorylates TRAF6 and MST4 upregulation reduces proinflammatory cytokine release (Jiao et al., 2015). Cereblon and Nod-like receptor C5 (NLRC5) interact with TRAF6 in contradictory fashions. Cereblon functions as a ubiquitin inhibitor by disturbing the TRAF6 zinc-finger ubiquitination domain (Min et al., 2016). NLRC5 is an endogenous regulator of the IKK complex and limits NF-κB translocation, but upon LPS stimulation NLRC5 is ubiquitinated by TRAF6. This suggests a secondary role for TRAF6 where it communicates outside of the paradigmatic inflammatory pathway to turn off regulators of downstream TLR signaling kinases (Cui et al., 2010; Meng et al., 2015). Furthermore, a recent study implicates the E3 ubiquitin ligase tripartite motif family 13 (Trim13) as a potentiator of TRAF6 ubiquitination and function. Trim13 knockdown inhibits TRAF6 K29-linked ubiquitination and NF-κB signaling more generally (Huang and Baek, 2017).

There are also several other proteins that may be important targets for TLR2 signaling modulation. Evidence suggests SHIP1 inhibits TBK1, a member of the TRIF-dependent signaling pathway (Gabhann et al., 2010; Thounaojam et al., 2014). Neutrophils derived from SHIP1^−/−^ mice are more susceptible to TLR2-mediated cytokine production as well as increased Akt activation (Strassheim et al., 2005). This result suggests that enhancing SHIP1 activity may reduce the proinflammatory response and reduce the ability for autophagy. SOCS1 also plays an inhibitory role restricting cytokine production through interactions with IRAK1 or TRAF6 (Nakagawa et al., 2002; West et al., 2006; Cardoso et al., 2012). Another possible therapeutic target is activated microglia/macrophage whey acidic protein (AMWAP), which is produced and released by microglia in neurotoxic conditions. Pretreatment of microglia with AMWAP prior to TLR2 stimulation reduces the mRNA expression of IL-6, iNOS, and TNFα, but the exact inhibitory interaction that prevents IκBα degradation is unclear (Aslanidis et al., 2015).

### Naturally occurring compounds

The identification and use of naturally occurring proteins and compounds has also proven fruitful in limiting inflammatory responses. Staphylococcal superantigen-like protein 3 (SSL3) is a product of *Staphylococcus aureus* that is used to evade the immune system through TLR2/1 dimer inhibition (Koymans et al., 2015). Further investigation with vaccinia virus proteins shows that A46R inhibits multiple TLR-adaptor interactions and A52R inhibits both TRAF6 and IRAK2. K1L and N1L are two additional vaccinia virus proteins that inhibit IκBα degradation and the downstream kinases TBK1 and IKKs respectively (Aravalli et al., 2008). These exogenous proteins, along with above endogenous protein regulators are shown in Figure 2. Other fungal products such as methylpenicinoline from *Penicillium* sp. (SF-5995) reduces both NF-κB translocation and the JNK-dependent signaling in BV2 microglia (Kim et al., 2014). Calcitriol, the active form of vitamin D, represses miR-155 after LPS stimulation and reduces NF-κB nuclear translocation (Chen et al., 2013b).

Plant-derived therapeutic compounds are among the most abundant in anti-inflammatory research. Multiple lignans and lignins suppress inflammation induced by TLR2- and TLR4-mediated pathways. These bioactive compounds are found in the Chinese medicinal plant *Schisandra chinensis* and are part of the dibenzocyclooctadiene family; schizandrin A (Song et al., 2016), schizandrin B (Giridharan et al., 2012; Zeng et al., 2012), and gomosins A/G/J/N inhibit microglial production of NO, TNFα, and IL-6. The gomosins act by increasing Nrf2 translocation and downregulating the MAPK pathways (Park et al., 2014). Pristimerin, an extract from *Tripterygium wildi*, reduces the release of NO and suppresses the protein and mRNA production of TNFα and IL-6 in BV2 microglia stimulated with LPS by inhibiting the IRAK1-TRAF6 interaction (Hui et al., 2018).

Various other herbal Chinese compounds also have potential for treating the neuroinflammatory response in synucleinopathies and their targets are listed in Table 1. The Chinese plant *Caesalpinia sappan* produces deoxysappone B, which inhibits the NF-κB and MAPK pathways and also reduces *in vitro* neuronal death in neuron-microglia cocultures (Zeng et al., 2015). Sparstolonin B is a product from *Sparganium stoloniferum* and it reduces IL-6 expression in a myocardial hypoxia model (Liu Q. et al., 2014) as well as TNFα following TLR2 stimulation in both RAW264.7 (Liang et al., 2015) and THP-1 cells by preventing the TLR2/1-MyD88 interaction (Liang et al., 2011). Lastly, a member of the coumarin family isolated from *Daphne odora*, named Daphnetin, decreases IL-6, TNFα, IL-1β, and NF-κB in models of anti-angiogenesis (Kumar et al., 2016), cerebral infarction (Liu et al., 2016), severe acute pancreatitis (Liu Z. Y. et al., 2014), and arthritis (Yao et al., 2011; Tu et al., 2012). One potential mode of action for Daphnetin may be through upregulation of A20, which modulates TRAF6 activity (Figure 2; Yu et al., 2014).

The *Cucurbitaceae* genus produces a number of compounds including cucurbitacins B, E, and I that reduce IL-6, TNFα, and IL-1β in microglia as well as induce Nrf2 nuclear translocation (Park et al., 2015). Cucurbitacin E also protects differentiated PC12 neurons from a PD model of MPTP-induced neurotoxicity (Arel-Dubeau et al., 2015). Similarly, kolaviron from the *Garcinia kola* plant, activates the Nrf2/ARE pathway and inhibits the release of IL-6 and TNFα in BV2 microglia following TLR4 stimulation (Onasanwo et al., 2016). Kolaviron is also neuroprotective and cardioprotective in rat models of oxidative stress (Olajide et al., 2017; Oyagbemi et al., 2017). In both *in vitro* and *in vivo* studies, Wang et al. demonstrate that pseudo-ginsenoside F11, derived from American ginseng, reduces microglial release of proinflammatory cytokines, inhibits Akt phosphorylation, and protects against neuronal death (Wang et al., 2014).

### Synthetic agents

The most diverse and robust class of TLR signaling inhibitors is the group of synthetic compounds found in Table 1. Starting at the origin of the signaling pathway, CU-CPT22 is known to be a TLR2/1 heterodimer antagonist with the ability to inhibit TLR2-mediated proinflammatory induction (Cheng et al., 2012; Daniele et al., 2015; Bock et al., 2016; Su et al., 2017). RSCL-0409, a gluco-disacharide, reduces TLR2 and TLR4 mediated TNFα release potentially through disruption of the TIR-adapter interaction, but the exact mechanism is not known (Kalluri et al., 2010). Similarly, C_16_H_15_NO_4_ (C29) and *ortho*-vanillin both block TLR2/1 signaling in murine macrophages via disruption of the MyD88 interaction with the TIR domain (Mistry et al., 2015). β-D-mannuronic acid (M2000) and its analog α-l-guluronic acid (G2013) are novel non-steroidal anti-inflammatory drugs (NSAIDs) currently being studied. In relation to oxidative stress, M2000 and G2013 non-significantly increase regulatory enzymes related to oxidative stress, but investigation of these two NSAIDs should not be immediately abandoned (Afraei et al., 2015; Mirshafiey et al., 2016; Hosseini et al., 2017). The mode of action for M2000 has been postulated as both a TLR2/TLR4 antagonist (Aletaha et al., 2017) and a miR-155 inhibitor (Pourgholi et al., 2017). Further investigations demonstrate M2000's ability to significantly decrease miR-146a, TRAF6, IRAK1, and NF-κB mRNA (Jahromi et al., 2017). These data indicate that oxidative stress markers may be the wrong outcome variables to assess the efficacy of these two NSAIDs and mediators of inflammation such as TLR2/1 signaling intermediates and the proinflammatory cytokines should be used instead. G2013 also appears to inhibit IRAK1/TRAF6 without modulation of miR-146a (Hajivalili et al., 2016; Nazeri et al., 2017), which could occur through regulation of Tollip mRNA transcript levels. The data indicates a high dose of G2013 lowers Tollip mRNA while also reducing NF-κB and IL-1β mRNA, which is counterintuitive with respect to the understanding of how Tollip functions as a negative regulator of IRAK1, and necessitates further investigation (Figure 2; Sharifi et al., 2017).

Many inhibitors target the cytosolic signaling proteins much like microRNA's. Dzamko et al. used a panel of inhibitors such as NG25, MRT67307, and BMS345541 which target TAK1 (Pauls et al., 2012), IKKε (Clark et al., 2011), and IKKβ respectively (Yang et al., 2006). All four successfully limit TLR2-mediated αSyn accumulation after TLR2 stimulation in SH-SY5Y cells, which demonstrates the efficacy of modulating the TLR2 pathway in both neurons and microglia (Dzamko et al., 2016). Another compound, MCAP, can inhibit IκBα phosphorylation and in turn reduce proinflammatory mRNA upregulation (Kim B.-W. et al., 2016). Lastly, Bay-11-708 a known inhibitor of NF-κB has been used to attenuate inflammation (Chen et al., 2013b; Matteucci et al., 2015).

Aside from direct inhibition of the TLR2 signaling pathway, there is also evidence supporting secondary roles for existing drugs and phagocytic inhibitors. The sartans are one such drug family that were first identified as angiotensin II receptor inhibitors. In a human monocyte model, candesartan downregulates TLR2 protein and mRNA, while also inhibiting NF-κB activation (Dasu et al., 2009). These results have been confirmed in primary microglia where candesartan reduced αSyn-mediated TNFα release (Daniele et al., 2015). Another drug, fasudil, which is a Rho kinase inhibitor, decreases TNFα and IL-1β, upregulates anti-oxidant/inflammatory molecules, Nrf2 and IL-10 (Liu et al., 2013; Zhao Y. F. et al., 2015), and protects TH^+^ neurons *in vivo* in a MPTP-model of PD (Li et al., 2017). And in an alternative approach to microglial inhibition, cyclic Arginine-Guanine-Aspartic Acid (cRGD), has been used to repress autophagy, which in turn reduces neuronal loss when co-cultured with TLR2-activated microglia (Neher et al., 2011). *In vivo* injection of cRGD reduced microglial-mediated neuronal phagocytosis in a mouse model of retinal degeneration (Zhao L. et al., 2015). These cRGD studies are intriguing insofar as they complicate the use of Akt as a therapeutic target. As discussed earlier, TLR2 activation and subsequent inhibition of Akt in neuronal cultures protected against cell death, but Neher et al. demonstrate TLR2 activation in conjunction with cRGD-mediated phagocytic inhibition protects neurons. This could be due to independent phagocytic pathways in microglia and neurons and we believe this necessitates further investigation.

All the above studies, and their respective drugs and targets (Table 1), provide a glimpse into the potential for discovering the next best course of treatment for synucleinopathies. Not all of the studies discussed have been verified in microglial models or more importantly *in vivo* models of disease. Further investigations and critical analyses of the mechanism of action for each potential inhibitor within the CNS are required before moving forward. We believe it is important to examine the potential choices available for TLR2 modulation—both the intended and potential unintended effects. For example, while antagonists of MyD88 signaling are available, the effects of inhibiting this widely used adaptor protein may prove to be a dangerous method for curtailing the microgliosis and neuroinflammation *in vivo*. Thus, we suggest a focus on the downstream effector proteins from the IRAKs to the IKKs as well as the Nrf2 and autophagocytic pathways in order to manage the proinflammatory response in synucleinopathies.

## Clinical trials and future therapeutics

The current landscape of clinical trial data is relatively small with regards to synucleinopathies and potential treatments involving modulation of microglia and the inflammatory response. While major therapeutics have not been discovered for treatments of synucleinopathies in humans, there have been advancements in characterization of neural inflammation. The peripheral benzodiazepine receptors (PBRs) are expressed on activated microglia and the PET PBR ligand [^11^C](R)-PK11195 provides a method for tracing microglial activation in patients. This diagnostic procedure has been validated multiple times (Gerhard et al., 2004, 2006a,b), and Ouchi et al. demonstrated that [^11^C](R)-PK11195 binding potential (BP) is inversely correlated with the dopamine transporter marker [^11^C]-CFT BP in the putamen (Ouchi et al., 2005). It is also interesting to note that that another group using [^11^C](R)-PK11195 PET described an inability to use the cerebellum as a BP control region, as has been done in Alzheimer's disease studies, and propose the need for more specific PBR tracers for synucleinopathies (Bartels et al., 2010). There is also an active clinical trial (NCT02702102) that is investigating the efficacy of [^11^C](R)-PBR28 as an alternative measure of microglial activation in DLB and PD with dementia (Kreisl et al., 2017).

Investigations into the use of NSAIDs and aspirin in neurodegenerative diseases initially seemed fruitful (Klegeris and McGeer, 2005). An epidemiological study demonstrated a decreased relative risk of developing PD if an individual took aspirin twice daily or regularly used NSAIDs on a weekly basis (Chen et al., 2003). The Bartels et al. study demonstrated the inefficacy of celecoxib in reducing microglial activation in PD patients (Bartels et al., 2010) and implementation of NSAIDs or rofecoxib, a COX-2 inhibitor, did not have any significant influence on AD progression (Aisen et al., 2003). Since both AD and PD are progressive and patients present with varying epigenetic and genetic backgrounds, the failure of these clinical trials could be due to the patient population selected. There has been a phase I/II trial for M2000 to assess the efficacy of this novel NSAID compared naproxen for ankylosing spondylitis. The therapeutic benefits was no different than naproxen, but M2000 did have fewer adverse effects that could make this drug more attractive for future PD related trials (Fattahi et al., 2017).

A number of clinical trials have attempted to modify disease pathogenesis or target the extracellular receptors, but few have been successful. For example, CEP-1347 is a JNK inhibitor used in early-stage PD patients in an attempt to enhance neuronal survival, but CEP-1347 had no effect in delaying the onset of more severe symptoms that required dopamine therapy (Wang et al., 2008). Further clinical studies using TLR4 antagonists, TAK-242 (Rice et al., 2010) and eritoran (E5564) (Bennett-Guerrero et al., 2007), both failed to significantly change cytokine levels or post-operative outcomes respectively, despite pre-clinical successes (Rossignol and Lynn, 2002; Ii et al., 2006). The failure of these two drugs may present a harbinger for the potential ineffectiveness of directly targeting the ligand binding domain of TLR2/1. Despite these failures ibudilast, an inhibitor of microglial activation via phosphodiesterase inhibition, shows significant effects in reducing the Subjective Opioid Withdrawal Scale scores of heroin users after ending daily heroin use (Cooper et al., 2016). A drug of this nature although separate from direct TLR2 pathway intervention could be a potential target for PD therapy, particularly in early stages of the diseases when microglial activation is accelerating but symptoms are not severe.

## Conclusions

The current body of literature surrounding the function and modulation of inflammation is continually growing and emphasizes how inflammation potentiates multiple disease states. The results described herein provide insight into the many ways inflammatory processes may result in continued neurodegeneration. While the disease initiating events in relation to synucleinopathies and other neurodegenerative diseases are still debated, it seems likely that the cyclicity of microglial activation and continued propagation of a proinflammatory neural environment is integral to the neuron loss that leads to the most severe synucleinopathy symptoms.

As previously discussed, there is reason to expect that not all findings from peripheral model systems will directly translate to studies of the CNS. But for some of the aforementioned compounds there is strong evidence to support their efficacy *in vitro* and in animal models, which may be translatable to human synucleinopathy patients. And for those strategies that are not immediately ready for clinical trials, such as microRNA or protein activity manipulation, there are now numerous avenues to investigate novel strategies to regulate these compounds in an effort to combat inflammation and microgliosis. The sheer wealth of possibilities brings hope toward finding successful pharmacological interventions to limit or even stop the disease progression of synucleinopathies.

## Author contributions

IC and KM-Z wrote this review. IC wrote the initial draft and prepared all the figures for this review.

### Conflict of interest statement

The authors declare that the research was conducted in the absence of any commercial or financial relationships that could be construed as a potential conflict of interest.

## References

[B1] Abend J. R. Ramalingam D. Kieffer-Kwon P. Uldrick T. S. Yarchoan R. Ziegelbauer J. M. (2012). Kaposi's sarcoma-associated herpesvirus microRNAs target IRAK1 and MYD88, two components of the toll-like receptor/interleukin-1R signaling cascade, to reduce inflammatory-cytokine expression. J. Virol. 86, 11663–11674. 10.1128/JVI.01147-12 22896623 PMC3486292

[B2] Afraei S. Azizi G. Zargar S. J. Sedaghat R. Mirshafiey A. (2015). New therapeutic approach by G2013 in experimental model of multiple sclerosis. Acta Neurol. Belg. 115, 259–266. 10.1007/s13760-014-0392-x 25388635

[B3] Aisen P. S. Schafer K. A. Grundman M. Pfeiffer E. Sano M. Davis K. L.. (2003). Effects of rofecoxib or naproxen vs placebo on Alzheimer disease progression: a randomized controlled trial. JAMA 289, 2819–2826. 10.1001/jama.289.21.2819 12783912

[B4] Akira S. Uematsu S. Takeuchi O. (2006). Pathogen recognition and innate immunity. Cell 124, 783–801. 10.1016/j.cell.2006.02.015 16497588

[B5] Aletaha S. Haddad L. Roozbehkia M. Bigdeli R. Asgary V. Mahmoudi M.. (2017). M2000 (β -D-mannuronic acid) as a novel antagonist for blocking the TLR2 and TLR4 downstream signalling pathway. Scand. J. Immunol. 85, 122–129. 10.1111/sji.12519 27943385

[B6] Alvarez-Erviti L. Couch Y. Richardson J. Cooper J. M. Wood M. J. A. (2011). α-synuclein release by neurons activates the inflammatory response in a microglial cell line. Neurosci. Res. 69, 337–342. 10.1016/j.neures.2010.12.020 21255620

[B7] Aravalli R. N. Hu S. Lokensgard J. R. (2008). Inhibition of toll-like receptor signaling in primary murine microglia. J. NeuroImmune Pharmacol. 3, 5–11. 10.1007/s11481-007-9097-8 18066668 PMC2585048

[B8] Arel-Dubeau A.-M. Longpré F. Bournival J. Tremblay C. Demers-Lamarche J. Haskova P.. (2015). Cucurbitacin E has neuroprotective properties and autophagic modulating activities on dopaminergic neurons. Oxid. Med. Cell. Longev. 2014:15. 10.1155/2014/425496 25574337 PMC4276330

[B9] Aslanidis A. Karlstetter M. Scholz R. Fauser S. Neumann H. Fried C.. (2015). Activated microglia/macrophage whey acidic protein (AMWAP) inhibits NFκB signaling and induces a neuroprotective phenotype in microglia. J. Neuroinflammation 12:77. 10.1186/s12974-015-0296-6 25928566 PMC4417279

[B10] Athanassiadou A. Voutsinas G. Psiouri L. Leroy E. Polymeropoulos M. H. Ilias A.. (1999). Genetic analysis of families with Parkinson disease that carry the Ala53Thr mutation in the gene encoding α-synuclein. Am. J. Hum. Genet. 65, 555–558. 10.1086/302486 10417297 PMC1377953

[B11] Baba M. Nakajo S. Tu P. H. Tomita T. Nakaya K. Lee V. M.. (1998). Aggregation of α-synuclein in Lewy bodies of sporadic Parkinson's disease and dementia with Lewy bodies. Am. J. Pathol. 152, 879–884. 9546347 PMC1858234

[B12] Babcock A. A. Wirenfeldt M. Holm T. Nielsen H. H. Dissing-Olesen L. Toft-Hansen H.. (2006). Toll-like receptor 2 signaling in response to brain injury: an innate bridge to neuroinflammation. J. Neurosci. 26, 12826–12837. 10.1523/JNEUROSCI.4937-05.2006 17151286 PMC6674840

[B13] Bachstetter A. D. Xing B. Van Eldik L. J. (2014). The p38alpha mitogen-activated protein kinase limits the CNS proinflammatory cytokine response to systemic lipopolysaccharide, potentially through an IL-10 dependent mechanism. J. Neuroinflammation 11:175. 10.1186/s12974-014-0175-6 25297465 PMC4193976

[B14] Baek D. Villén J. Shin C. Camargo F. D. Gygi S. P. Bartel D. P. (2008). The impact of microRNAs on protein output. Nature 455, 64–71. 10.1038/nature07242 18668037 PMC2745094

[B15] Bala S. Marcos M. Kodys K. Csak T. Catalano D. Mandrekar P. (2011). Up-regulation of microRNA-155 in macrophages contributes to increased Tumor Necrosis Factor α (TNFα) production via increased mRNA half-life in alcoholic liver disease. J. Biol. Chem. 286, 1436–1444. 10.1074/jbc.M110.145870 21062749 PMC3020752

[B16] Bartels A. L. Willemsen A. T. M. Doorduin J. de Vries E. F. J. Dierckx R. A. Leenders K. L. (2010). [11C]-PK11195 PET: quantification of neuroinflammation and a monitor of anti-inflammatory treatment in Parkinson's disease? Park. Relat. Disord. 16, 57–59. 10.1016/j.parkreldis.2009.05.005 19487152

[B17] Bennett-Guerrero E. Grocott H. P. Levy J. H. Stierer K. A. Hogue C. W. Cheung A. T.. (2007). A Phase II, double-blind, placebo-controlled, ascending-dose study of eritoran (E5564), a lipid a antagonist, in patients undergoing cardiac surgery with cardiopulmonary bypass. Anesth. Analg. 104, 378–383. 10.1213/01.ane.0000253501.07183.2a 17242095

[B18] Béraud D. Hathaway H. A. Trecki J. Chasovskikh S. Johnson D. A. Johnson J. A.. (2013). Microglial activation and antioxidant responses induced by the Parkinson's disease protein α-synuclein. J. Neuroimmune Pharmacol. 8, 94–117. 10.1007/s11481-012-9401-0 23054368 PMC3582877

[B19] Bernheimer H. Birkmayer W. Hornykiewicz O. Jellinger K. Seitelberger F. (1973). Brain dopamine and the syndromes of Parkinson and Huntington: clinical, morphological and neurochemical correlations. J. Neurol. Sci. 20, 415–455. 10.1016/0022-510X(73)90175-5 4272516

[B20] Block M. L. Zecca L. Hong J. S. (2007). Microglia-mediated neurotoxicity: uncovering the molecular mechanisms. Nat. Rev. Neurosci. 8, 57–69. 10.1038/nrn2038 17180163

[B21] Bock S. Murgueitio M. S. Wolber G. Weindl G. (2016). Acute myeloid leukaemia-derived Langerhans-like cells enhance Th1 polarization upon TLR2 engagement. Pharmacol. Res. 105, 44–53. 10.1016/j.phrs.2016.01.016 26794428

[B22] Braak H. Del Tredici K. (2008). Invited Article: nervous system pathology in sporadic Parkinson disease. Neurology 70, 1916–1925. 10.1212/01.wnl.0000312279.49272.9f 18474848

[B23] Braak H. Del Tredici K. (2017). neuropathological staging of brain pathology in sporadic Parkinson's disease: separating the wheat from the chaff. J. Parkinsons. Dis. 7, S73–S87. 10.3233/JPD-179001 28282810 PMC5345633

[B24] Braak H. Del Tredici K. Rüb U. De Vos R. A. I. Jansen Steur E. N. H. Braak E. (2003). Staging of brain pathology related to sporadic Parkinson's disease. Neurobiol. Aging 24, 197–211. 10.1016/S0197-4580(02)00065-9 12498954

[B25] Breydo L. Wu J. W. Uversky V. N. (2012). α-Synuclein misfolding and Parkinson's disease. Biochim. Biophys. Acta Mol. Basis Dis. 1822, 261–285. 10.1016/j.bbadis.2011.10.002 22024360

[B26] Brown V. Brown R. A. Ozinsky A. Hesselberth J. R. Fields S. (2006). Binding specificity of toll-like receptor cytoplasmic domains. Eur. J. Immunol. 36, 742–753. 10.1002/eji.200535158 16482509 PMC2762736

[B27] Brun P. Giron M. C. Qesari M. Porzionato A. Caputi V. Zoppellaro C.. (2013). Toll-like receptor 2 regulates intestinal inflammation by controlling integrity of the enteric nervous system. Gastroenterology 145, 1323–1333. 10.1053/j.gastro.2013.08.047 23994200

[B28] Buwitt-Beckmann U. Heine H. Wiesmüller K. H. Jung G. Brock R. Akira S.. (2006). TLR1- and TLR6-independent recognition of bacterial lipopeptides. J. Biol. Chem. 281, 9049–9057. 10.1074/jbc.M512525200 16455646

[B29] Cardoso A. L. Guedes J. R. Pereira de Almeida L. Pedroso de Lima M. C. (2012). miR-155 modulates microglia-mediated immune response by down-regulating SOCS-1 and promoting cytokine and nitric oxide production. Immunology 135, 73–88. 10.1111/j.1365-2567.2011.03514.x 22043967 PMC3246654

[B30] Chen G. Nunez G. (2010). Sterile inflammation: sensing and reacting to damage. Nat. Rev. Immunol. 10, 826–837. 10.1038/nri2873 21088683 PMC3114424

[B31] Chen H. Zhang S. M. Hernan M. A. Schwarzschild M. Willet W. Colditz G. (2003). Nonsteroidal anti-inflammatory drugs and the risk of Parkinson disease. Arch. Neurol. 28, 193–196. 10.1001/archneur.60.8.1059 12925360

[B32] Chen Y. Chen J. Wang H. Shi J. Wu K. Liu S.. (2013a). HCV-induced miR-21 contributes to evasion of host immune system by targeting MyD88 and IRAK1. PLoS Pathog. 9:e1003248. 10.1371/journal.ppat.1003248 23633945 PMC3635988

[B33] Chen Y. Liu W. Sun T. Huang Y. Wang Y. Deb D. K.. (2013b). 1,25-Dihydroxyvitamin D promotes negative feedback regulation of TLR signaling via targeting microRNA-155-SOCS1 in macrophages. J. Immunol. 190, 3687–3695. 10.4049/jimmunol.1203273 23436936 PMC3608760

[B34] Cheng K. Wang X. Zhang S. Yin H. (2012). Discovery of small-molecule inhibitors of the TLR1/TLR2 complex. Angew. Chemie Int. Ed. 51, 12246–12249. 10.1002/anie.201204910 22969053 PMC3510333

[B35] Chung Y. C. Ko H. W. Bok E. Huh S. H. Nam J. H. Jin B. K. (2010). The role of neuroinflammation on the pathogenesis of Parkinson's disease. BMB Rep. 43, 225–232. 10.5483/BMBRep.2010.43.4.225 20423606

[B36] Clark K. Peggie M. Plater L. Sorcek R. J. Young E. R. R. Madwed J. B.. (2011). Novel cross-talk within the IKK family controls innate immunity. Biochem. J. 434, 93–104. 10.1042/BJ20101701 21138416

[B37] Conway K. A. Harper J. D. Lansbury P. T. (1998). Accelerated *in vitro* fibril formation by a mutant α-synuclein linked to early-onset Parkinson disease. Nat. Med. 4, 1318–1320. 10.1038/3311 9809558

[B38] Conway K. A. Harper J. D. Lansbury P. T. (2000). Fibrils formed *in vitro* from α-synuclein and two mutant forms linked to Parkinson's disease are typical amyloid. Biochemistry 39, 2552–2563. 10.1021/bi991447r 10704204

[B39] Conway K. A. Rochet J. C. Bieganski R. M. Lansbury P. T. (2001). Kinetic stabilization of the α-synuclein protofibril by a dopamine-α-synuclein adduct. Science 294, 1346–1349. 10.1126/science.1063522 11701929

[B40] Cooper Z. D. Johnson K. W. Pavlicova M. Glass A. Vosburg S. K. Sullivan M. A.. (2016). The effects of ibudilast, a glial activation inhibitor, on opioid withdrawal symptoms in opioid-dependent volunteers. Addict. Biol. 21, 895–903. 10.1111/adb.12261 25975386 PMC4644513

[B41] Cui J. Zhu L. Xia X. Wang H. Y. Legras X. Hong J.. (2010). NLRC5 negatively regulates the NF-κB and type I interferon signaling pathways. Cell 141, 483–496. 10.1016/j.cell.2010.03.040 20434986 PMC3150216

[B42] Daniele S. G. Béraud D. Davenport C. Cheng K. Yin H. Maguire-Zeiss K. A. (2015). Activation of MyD88-dependent TLR1/2 signaling by misfolded α-synuclein, a protein linked to neurodegenerative disorders. Sci. Signal. 8:ra45. 10.1126/scisignal.2005965 25969543 PMC4601639

[B43] Danzer K. M. Haasen D. Karow A. R. Moussaud S. Habeck M. Giese A.. (2007). Different species of α-synuclein oligomers induce calcium influx and seeding. J. Neurosci. 27, 9220–9232. 10.1523/JNEUROSCI.2617-07.2007 17715357 PMC6672196

[B44] Dasu M. R. Riosvelasco A. C. Jialal I. (2009). Candesartan inhibits Toll-like receptor expression and activity both *in vitro* and *in vivo*. Atherosclerosis 202, 76–83. 10.1016/j.atherosclerosis.2008.04.010 18495130 PMC2676176

[B45] Davalos D. Grutzendler J. Yang G. Kim J. V. Zuo Y. Jung S.. (2005). ATP mediates rapid microglial response to local brain injury *in vivo*. Nat. Neurosci. 8, 752–758. 10.1038/nn1472 15895084

[B46] de Lau L. M. L. Breteler M. M. B. (2006). Epidemiology of Parkinson's disease. Lancet. Neurol. 5, 525–535. 10.1016/S1474-4422(06)70471-9 16713924

[B47] Deleersnijder A. Gerard M. Debyser Z. Baekelandt V. (2013). The remarkable conformational plasticity of α-synuclein: blessing or curse? Trends Mol. Med. 19, 368–377. 10.1016/j.molmed.2013.04.002 23648364

[B48] de Sousa A. A. Reis R. Bento-Torres J. Trévia N. de Lins N. A. A. Passos A.. (2011). Influence of enriched environment on viral encephalitis outcomes: behavioral and neuropathological changes in albino Swiss mice. PLoS ONE 6:e15597. 10.1371/journal.pone.0015597 21264301 PMC3019164

[B49] Desplats P. Lee H.-J. Bae E.-J. Patrick C. Rockenstein E. Crews L.. (2009). Inclusion formation and neuronal cell death through neuron-to-neuron transmission of α-synuclein. Proc. Natl. Acad. Sci. U.S.A. 106, 13010–13015. 10.1073/pnas.0903691106 19651612 PMC2722313

[B50] Dettmer U. Newman A. J. Luth E. S. Bartels T. Selkoe D. (2013). *In vivo* cross-linking reveals principally oligomeric forms of α-synuclein and β-synuclein in neurons and non-neural cells. J. Biol. Chem. 288, 6371–6385. 10.1074/jbc.M112.403311 23319586 PMC3585072

[B51] Dev K. K. Hofele K. Barbieri S. Buchman V. L. Van Der Putten H. (2003). Part II: α-synuclein and its molecular pathophysiological role in neurodegenerative disease. Neuropharmacology 45, 14–44. 10.1016/S0028-3908(03)00140-0 12814657

[B52] Didierlaurent A. Brissoni B. Velin D. Aebi N. Tardivel A. Käslin E.. (2006). Tollip regulates proinflammatory responses to interleukin-1 and lipopolysaccharide. Mol. Cell. Biol. 26, 735–742. 10.1128/MCB.26.3.735-742.2006 16428431 PMC1347014

[B53] Ding T. T. Lee S. Rochet J.-C. Lansbury P. T. (2002). Annular α-synuclein protofibrils are produced when spherical protofibrils are incubated in solution or bound to brain-derived membranes ^†^. Biochemistry 41, 10209–10217. 10.1021/bi020139h 12162735

[B54] Doorn K. J. Moors T. Drukarch B. van de Berg W. D. J. Lucassen P. J. van Dam A.-M. (2014). Microglial phenotypes and toll-like receptor 2 in the substantia nigra and hippocampus of incidental Lewy body disease cases and Parkinson's disease patients. Acta Neuropathol. Commun. 2, 1–17. 10.1186/s40478-014-0090-1 25099483 PMC4224021

[B55] Downer E. J. Johnston D. G. W. Lynch M. A. (2013). Differential role of Dok1 and Dok2 in TLR2-induced inflammatory signaling in glia. Mol. Cell. Neurosci. 56, 148–158. 10.1016/j.mcn.2013.04.007 23659921

[B56] Drouin-Ouellet J. St-Amour I. Saint-Pierre M. Lamontagne-Proulx J. Kriz J. Barker R. A.. (2015). Toll-like receptor expression in the blood and brain of patients and a mouse model of Parkinson's disease. Int. J. Neuropsychopharmacol. 18, 1–11. 10.1093/ijnp/pyu103 25522431 PMC4438545

[B57] Dzamko N. Gysbers A. Perera G. Bahar A. Shankar A. Gao J.. (2016). Toll-like receptor 2 is increased in neurons in Parkinson's disease brain and may contribute to α-synuclein pathology. Acta Neuropathol. 133, 1–17. 10.1007/s00401-016-1648-8 27888296 PMC5250664

[B58] Emmanouilidou E. Elenis D. Papasilekas T. Stranjalis G. Gerozissis K. Ioannou P. C.. (2011). Assessment of α-synuclein secretion in mouse and human brain parenchyma. PLoS ONE 6:e22225. 10.1371/journal.pone.0022225 21779395 PMC3136497

[B59] Fahn S. (2003). Description of Parkinson's disease as a clinical syndrome. Ann. N.Y. Acad. Sci. 991, 1–14. 10.1111/j.1749-6632.2003.tb07458.x 12846969

[B60] Fattahi M. J. Jamshidi A. R. Mahmoudi M. Vojdanian M. Yekaninejad M. S. Jafarnezhad-Ansariha F.. (2017). Evaluation of the efficacy and safety of β-d-mannuronic acid in patients with ankylosing spondylitis: a 12-week randomized, placebo-controlled, phase I/II clinical trial. Int. Immunopharmacol. 54, 112–117. 10.1016/j.intimp.2017.11.003 29127910

[B61] Fellner L. Irschick R. Schanda K. Reindl M. Klimaschewski L. Poewe W.. (2013). Toll-like receptor 4 is required for α-synuclein dependent activation of microglia and astroglia. Glia 61, 349–360. 10.1002/glia.22437 23108585 PMC3568908

[B62] Ferrari C. C. Pott Godoy M. C. Tarelli R. Chertoff M. Depino A. M. Pitossi F. J. (2006). Progressive neurodegeneration and motor disabilities induced by chronic expression of IL-1β in the substantia nigra. Neurobiol. Dis. 24, 183–193. 10.1016/j.nbd.2006.06.013 16901708

[B63] Fujiwara H. Hasegawa M. Dohmae N. Kawashima A. Masliah E. Goldberg M. S.. (2002). α-Synuclein is phosphorylated in synucleinopathy lesions. Nat. Cell Biol. 4, 160–164. 10.1038/ncb748 11813001

[B64] Gabhann J. N. Higgs R. Brennan K. Thomas W. Damen J. E. Ben Larbi N.. (2010). Absence of SHIP-1 results in constitutive phosphorylation of tank-binding kinase 1 and enhanced TLR3-dependent IFN-β production. J. Immunol. 184, 2314–2320. 10.4049/jimmunol.0902589 20100929

[B65] Gao H.-M. Kotzbauer P. T. Uryu K. Leight S. Trojanowski J. Q. Lee V. M.-Y. (2008). Neuroinflammation and oxidation/nitration of α-synuclein linked to dopaminergic neurodegeneration. J. Neurosci. 28, 7687–7698. 10.1523/JNEUROSCI.0143-07.2008 18650345 PMC2702093

[B66] Gerhard A. Pavese N. Hotton G. Turkheimer F. Es M. Hammers A. (2006a). *In vivo* imaging of microglial activation with [11C](R)-PK11195 PET in idiopathic Parkinson's disease. Neurobiol. Dis. 21, 404–412. 10.1016/j.nbd.2005.08.002 16182554

[B67] Gerhard A. Trender-Gerhard I. Turkheimer F. Quinn N. P. Bhatia K. P. Brooks D. J. (2006b). *In vivo* imaging of microglial activation with [11C](R)-PK11195 PET in progressive supranuclear palsy. Mov. Disord. 21, 89–93. 10.1002/mds.20668 16108021

[B68] Gerhard A. Watts J. Trender-Gerhard I. Turkheimer F. Banati R. B. Bhatia K. (2004). *In vivo* imaging of microglial activation with [11C](R -PK11195 PET in corticobasal degeneration. Mov. Disord. 19, 1221–1226. 10.1002/mds.20162 15390000

[B69] Giridharan V. V. Thandavarayan R. A. Bhilwade H. N. Ko K. M. Watanabe K. Konishi T. (2012). Schisandrin B, attenuates cisplatin-induced oxidative stress, genotoxicity and neurotoxicity through modulating NF-κB pathway in mice. Free Radic. Res. 46, 50–60. 10.3109/10715762.2011.638291 22059853

[B70] Grabert K. Michoel T. Karavolos M. H. Clohisey S. Baillie J. K. Stevens M. P.. (2016). Microglial brain region-dependent diversity and selective regional sensitivities to aging. Nat. Neurosci. 19, 504–516. 10.1038/nn.4222 26780511 PMC4768346

[B71] Guo H. Chen Y. Hu X. Qian G. Ge S. Zhang J. (2013). The regulation of Toll-like receptor 2 by miR-143 suppresses the invasion and migration of a subset of human colorectal carcinoma cells. Mol. Cancer 12:77. 10.1186/1476-4598-12-77 23866094 PMC3750391

[B72] Gustot A. Gallea J. I. Sarroukh R. Celej M. S. Ruysschaert J.-M. Raussens V. (2015). Amyloid fibrils are the molecular trigger of inflammation in Parkinson's disease. Biochem. J. 471, 323–333. 10.1042/BJ20150617 26272943

[B73] Guven-Maiorov E. Keskin O. Gursoy A. Nussinov R. (2015). A structural view of negative regulation of the toll-like receptor-mediated inflammatory pathway. Biophys. J. 109, 1214–1220. 10.1016/j.bpj.2015.06.048 26276688 PMC4576153

[B74] Hajivalili M. Pourgholi F. Majidi J. Aghebati-Maleki L. Movassaghpour A. A. Samadi Kafil H.. (2016). G2013 modulates TLR4 signaling pathway in IRAK-1 and TARF-6 dependent and miR-146a independent manner. Cell. Mol. Biol. (Noisy-le-grand). 62, 1–5. 10.14715/cmb/2016.62.4.1 27188726

[B75] Hanisch U.-K. (2013). Functional diversity of microglia - how heterogeneous are they to begin with? Front. Cell. Neurosci. 7:65. 10.3389/fncel.2013.00065 23717262 PMC3653062

[B76] Hashimoto M. Hsu L. J. Xia Y. Takeda A. Sisk A. Sundsmo M.. (1999). Oxidative stress induces amyloid-like aggregate formation of NACP/α-synuclein *in vitro*. Neuroreport 10, 717–721. 10.1097/00001756-199903170-00011 10208537

[B77] He Q. Wang Q. Yuan C. Wang Y. (2017). Downregulation of miR-7116-5p in microglia by MPP+ sensitizes TNF-α production to induce dopaminergic neuron damage. Glia 65, 1251–1263. 10.1002/glia.23153 28543680

[B78] Horng T. Barton G. M. Flavell R. A. Medzhitov R. (2002). The adaptor molecule TIRAP provides signalling specificity for Toll-like receptors. Nature 420, 329–333. 10.1038/nature01180 12447442

[B79] Hosseini S. Abdollahi M. Azizi G. Fattahi M. J. Rastkari N. Zavareh F. T.. (2017). Anti-aging effects of M2000 (β-D-mannuronic acid) as a novel immunosuppressive drug on the enzymatic and non-enzymatic oxidative stress parameters in an experimental model. J. Basic Clin. Physiol. Pharmacol. 28, 249–255. 10.1515/jbcpp-2016-0092 28207414

[B80] Huang B. Baek S. (2017). Trim13 potentiates toll-like receptor 2-mediated nuclear factor κB activation via K29-linked polyubiquitination of tumor necrosis factor receptor-associated factor 6. Mol. Pharmacol. 91, 307–316. 10.1124/mol.116.106716 28087809

[B81] Hui B. Zhang L. Zhou Q. Hui L. (2018). Pristimerin inhibits LPS-triggered neurotoxicity in BV-2 microglia cells through modulating IRAK1/TRAF6/TAK1-mediated NF-κB and AP-1 signaling pathways *in vitro*. Neurotox. Res. 33, 268–283. 10.1007/s12640-017-9837-3 29119451

[B82] Humbert-Claude M. Duc D. Dwir D. Thieren L. Sandström von Tobel J. Begka C.. (2016). Tollip, an early regulator of the acute inflammatory response in the substantia nigra. J. Neuroinflammation 13:303. 10.1186/s12974-016-0766-5 27927222 PMC5142340

[B83] Ii M. Matsunaga N. Hazeki K. Nakamura K. Takashima K. Seya T.. (2006). A novel cyclohexene derivative, ethyl (6R)-6-[N-(2-Chloro-4-fluorophenyl)sulfamoyl]cyclohex-1-ene-1-carboxylate (TAK-242), selectively inhibits toll-like receptor 4-mediated cytokine production through suppression of intracellular signaling. Mol. Pharmacol. 69, 1288–1295. 10.1124/mol.105.019695 16373689

[B84] Innamorato N. G. Lastres-Becker I. Cuadrado A. (2009). Role of microglial redox balance in modulation of neuroinflammation. Curr. Opin. Neurol. 22, 308–314. 10.1097/WCO.0b013e32832a3225 19359988

[B85] Innamorato N. G. Rojo A. I. García-Yagüe A. J. Yamamoto M. de Ceballos M. L. Cuadrado A. (2008). The transcription factor Nrf2 is a therapeutic target against brain inflammation. J. Immunol. 181, 680–689. 10.4049/jimmunol.181.1.680 18566435

[B86] Jahromi S. S. M. Jamshidi M. M. Farazmand A. Aghazadeh Z. Yousefi M. Mirshafiey A. (2017). Pharmacological effects of β-D-mannuronic acid (M2000) on miR-146a, IRAK1, TRAF6 and NF-κB gene expression, as target molecules in inflammatory reactions. Pharmacol. Rep. 69, 479–484. 10.1016/j.pharep.2017.01.021 28324845

[B87] Jana M. Palencia C. A. Pahan K. (2008). Fibrillar amyloid-β peptides activate microglia via TLR2: implications for Alzheimer's disease. J. Immunol. 181, 7254–7262. 10.4049/jimmunol.181.10.7254 18981147 PMC2701549

[B88] Jang A. Lee H. J. Suk J. E. Jung J. W. Kim K. P. Lee S. J. (2010). Non-classical exocytosis of α-synuclein is sensitive to folding states and promoted under stress conditions. J. Neurochem. 113, 1263–1274. 10.1111/j.1471-4159.2010.06695.x 20345754

[B89] Jiao S. Zhang Z. Li C. Huang M. Shi Z. Wang Y.. (2015). The kinase MST4 limits inflammatory responses through direct phosphorylation of the adaptor TRAF6. Nat. Immunol. 16, 246–257. 10.1038/ni.3097 25642822

[B90] Jin M. S. Kim S. E. Heo J. Y. Lee M. E. Kim H. M. Paik S. G.. (2007). Crystal structure of the TLR1-TLR2 heterodimer induced by binding of a tri-acylated lipopeptide. Cell 130, 1071–1082. 10.1016/j.cell.2007.09.008 17889651

[B91] Johnson G. L. Lapadat R. (2002). Mitogen-activated protein kinase pathways mediated by ERK, JNK, and p38 protein kinases. Science 298, 1911–1912. 10.1126/science.1072682 12471242

[B92] Jurkin J. Schichl Y. M. Koeffel R. Bauer T. Richter S. Konradi S.. (2010). miR-146a is differentially expressed by myeloid dendritic cell subsets and desensitizes cells to TLR2-dependent activation. J. Immunol. 184, 4955–4965. 10.4049/jimmunol.0903021 20375304

[B93] Kacimi R. Giffard R. G. Yenari M. A. (2011). Endotoxin-activated microglia injure brain derived endothelial cells via NF-κB, JAK-STAT and JNK stress kinase pathways. J. Inflamm. (Lond). 8:7. 10.1186/1476-9255-8-7 21385378 PMC3061894

[B94] Kalluri M. D. Datla P. Bellary A. Basha K. Sharma A. Sharma A.. (2010). Novel synthetic gluco-disaccharide RSCL-0409 - a lipopolysaccharide-induced Toll-like receptor-mediated signalling antagonist. FEBS J. 277, 1639–1652. 10.1111/j.1742-4658.2010.07589.x 20180845

[B95] Karpinar D. P. Balija M. B. G. Kügler S. Opazo F. Rezaei-Ghaleh N. Wender N. (2009). Pre-fibrillar alpha-synuclein variants with impaired α-structure increase neurotoxicity in Parkinson's disease models. EMBO J. 28, 3256–3268. 10.1038/emboj.2009.257 19745811 PMC2771093

[B96] Kawai T. Akira S. (2007). Signaling to NF-κB by toll-like receptors. Trends Mol. Med. 13, 460–469. 10.1016/j.molmed.2007.09.002 18029230

[B97] Kawai T. Akira S. (2010). The role of pattern-recognition receptors in innate immunity: update on Toll-like receptors. Nat. Immunol. 11, 373–384. 10.1038/ni.1863 20404851

[B98] Kim B.-W. More S. V. Yun Y.-S. Ko H.-M. Kwak J.-H. Lee H.. (2016). A novel synthetic compound MCAP suppresses LPS-induced murine microglial activation in vitro via inhibiting NF-kB and p38 MAPK pathways. Acta Pharmacol. Sin. 37, 334–343. 10.1038/aps.2015.138 26838070 PMC4775849

[B99] Kim C. C. Nakamura M. C. Hsieh C. L. (2016). Brain trauma elicits non-canonical macrophage activation states. J. Neuroinflammation 13:117. 10.1186/s12974-016-0581-z 27220367 PMC4879757

[B100] Kim C. Ho D. Suk J. You S. Michael S. Kang J.. (2013). Neuron-released oligomeric α-synuclein is an endogenous agonist of TLR2 for paracrine activation of microglia. Nat. Commun. 4:1562. 10.1038/ncomms2534 23463005 PMC4089961

[B101] Kim C. Lee H.-J. Masliah E. Lee S.-J. (2016). Non-cell-autonomous neurotoxicity of α-synuclein through microglial toll-like receptor 2. Exp. Neurobiol. 25, 113–119. 10.5607/en.2016.25.3.113 27358579 PMC4923355

[B102] Kim C. Rockenstein E. Spencer B. Kim H. K. Adame A. Trejo M.. (2015). Antagonizing neuronal toll-like receptor 2 prevents synucleinopathy by activating autophagy. Cell Rep. 13, 771–782. 10.1016/j.celrep.2015.09.044 26489461 PMC4752835

[B103] Kim D. C. Lee H. S. Ko W. Lee D. S. Sohn J. H. Yim J. H.. (2014). Anti-inflammatory effect of methylpenicinoline from a marine isolate of *Penicillium* sp. (SF-5995): inhibition of NF-κB and MAPK pathways in lipopolysaccharide-induced RAW264.7 macrophages and BV2 microglia. Molecules 19, 18073–18089. 10.3390/molecules191118073 25379644 PMC6271136

[B104] Klegeris A. McGeer P. (2005). Non-steroidal anti-inflammatory drugs (NSAIDs) and other anti- inflammatory agents in the treatment of neurodegenerative disease. Curr. Alzheimer Res. 2, 355–365. 10.2174/1567205054367883 15974901

[B105] Knott C. Stern G. Wilkin G. P. (2000). Inflammatory regulators in Parkinson's disease: iNOS, lipocortin-1, and cyclooxygenases-1 and−2. Mol. Cell. Neurosci. 16, 724–739. 10.1006/mcne.2000.0914 11124893

[B106] Koh K. Kim J. Jang Y. J. Yoon K. Cha Y. Lee H. J.. (2011). Transcription factor Nrf2 suppresses LPS-induced hyperactivation of BV-2 microglial cells. J. Neuroimmunol. 233, 160–167. 10.1016/j.jneuroim.2011.01.004 21349591

[B107] Koymans K. J. Feitsma L. J. Brondijk T. H. C. Aerts P. C. Lukkien E. Lossl P.. (2015). Structural basis for inhibition of TLR2 by staphylococcal superantigen-like protein 3 (SSL3). Proc. Natl. Acad. Sci. U.S.A. 112, 11018–11023. 10.1073/pnas.1502026112 26283364 PMC4568226

[B108] Kreisl W. C. Kats B. Kats R. (2017). Imaging inflammation in patients with diffuse lewy body disease. Available Online at: https://clinicaltrials.gov/ct2/ (Identification Number NCT02702102).

[B109] Krichevsky A. M. King K. S. Donahue C. P. Khrapko K. Kosik K. S. (2003). A microRNA array reveals extensive regulation of microRNAs during brain development. RNA 9, 1274–1281. 10.1261/rna.5980303 13130141 PMC1370491

[B110] Krüger R. Kuhn W. Müller T. Woitalla D. Graeber M. Kösel S.. (1998). Ala30Pro mutation in the gene encoding α-synuclein in Parkinson's disease. Nat. Genet. 18, 106–108. 9462735 10.1038/ng0298-106

[B111] Kumar A. Sunita P. Jha S. Pattanayak S. P. (2016). Daphnetin inhibits TNF-α and VEGF-induced angiogenesis through inhibition of the IKKs/IκBα/NF-κB, Src/FAK/ERK1/2 and Akt signalling pathways. Clin. Exp. Pharmacol. Physiol. 43, 939–950. 10.1111/1440-1681.12608 27297262

[B112] Lafon M. Megret F. Lafage M. Prehaud C. (2006). The innate immune facet of brain: human neurons express TLR-3 and sense viral dsRNA. J. Mol. Neurosci. 29, 185–194. 10.1385/JMN:29:3:185 17085778

[B113] Lai B. C. L. Marion S. A. Teschke K. Tsui J. K. C. (2002). Occupational and environmental risk factors for Parkinson's disease. Parkinsonism Relat. Disord. 8, 297–309. 10.1016/S1353-8020(01)00054-2 15177059

[B114] Lai L. Song Y. Liu Y. Chen Q. Han Q. Chen W.. (2013). MicroRNA-92a negatively regulates toll-like receptor (TLR)-triggered inflammatory response in macrophages by targeting MKK4 kinase. J. Biol. Chem. 288, 7956–7967. 10.1074/jbc.M112.445429 23355465 PMC3597832

[B115] Landais I. Pelton C. Streblow D. DeFilippis V. McWeeney S. Nelson J. A. (2015). Human cytomegalovirus miR-UL112-3p targets TLR2 and modulates the TLR2/IRAK1/NFκB signaling pathway. PLoS Pathog. 11:e1004881. 10.1371/journal.ppat.1004881 25955717 PMC4425655

[B116] Lashuel H. A. Overk C. R. Oueslati A. Masliah E. (2012). The many faces of α-synuclein: from structure and toxicity to therapeutic target. Nat. Rev. Neurosci. 14, 38–48. 10.1038/nrn3406 23254192 PMC4295774

[B117] Lee E.-J. Woo M.-S. Moon P.-G. Baek M.-C. Choi I.-Y. Kim W.-K.. (2010). α-Synuclein activates microglia by inducing the expressions of matrix metalloproteinases and the subsequent activation of protease-activated receptor-1. J. Immunol. 185, 615–623. 10.4049/jimmunol.0903480 20511551

[B118] Lee H.-J. Patel S. Lee S.-J. (2005). Intravesicular localization and exocytosis of α-synuclein and its aggregates. J. Neurosci. 25, 6016–6024. 10.1523/JNEUROSCI.0692-05.2005 15976091 PMC6724798

[B119] Lee S. Suk K. (2007). Heme oxygenase-1 mediates cytoprotective effects of immunostimulation in microglia. Biochem. Pharmacol. 74, 723–729. 10.1016/j.bcp.2007.06.016 17632083

[B120] Lehnardt S. Massillon L. Follett P. Jensen F. E. Ratan R. Rosenberg P. A.. (2003). Activation of innate immunity in the CNS triggers neurodegeneration through a Toll-like receptor 4-dependent pathway. Proc. Natl. Acad. Sci. U.S.A. 100, 8514–8519. 10.1073/pnas.1432609100 12824464 PMC166260

[B121] Li W. Khor T. O. Xu C. Shen G. Jeong W. S. Yu S.. (2008). Activation of Nrf2-antioxidant signaling attenuates NFκB-inflammatory response and elicits apoptosis. Biochem. Pharmacol. 76, 1485–1489. 10.1016/j.bcp.2008.07.017 18694732 PMC2610259

[B122] Li Y.-H. Yu J.-W. Xi J.-Y. Yu W.-B. Liu J.-C. Wang Q.. (2017). Fasudil enhances therapeutic efficacy of neural stem cells in the mouse model of MPTP-induced Parkinson's disease. Mol. Neurobiol. 54, 5400–5413. 10.1007/s12035-016-0027-8 27590141

[B123] Liang Q. Dong S. Lei L. Liu J. Zhang J. Li J.. (2015). Protective effects of Sparstolonin B, a selective TLR2 and TLR4 antagonist, on mouse endotoxin shock. Cytokine 75, 302–309. 10.1016/j.cyto.2014.12.003 25573805 PMC4950682

[B124] Liang Q. Wu Q. Jiang J. Duan J. Wang C. Smith M. D.. (2011). Characterization of sparstolonin B, a chinese herb-derived compound, as a selective toll-like receptor antagonist with potent anti-inflammatory properties. J. Biol. Chem. 286, 26470–26479. 10.1074/jbc.M111.227934 21665946 PMC3143611

[B125] Liang S. Hosur K. B. Lu S. Nawar H. F. Weber B. R. Tapping R. I.. (2009). Mapping of a microbial protein domain involved in binding and activation of the TLR2/TLR1 heterodimer. J. Immunol. 182, 2978–2985. 10.4049/jimmunol.0803737 19234193 PMC2648068

[B126] Lin S.-C. Lo Y.-C. Wu H. (2010). Helical assembly in the MyD88-IRAK4-IRAK2 complex in TLR/IL-1R signalling. Nature 465, 885–890. 10.1038/nature09121 20485341 PMC2888693

[B127] Liu C. Li Y. Yu J. Feng L. Hou S. Liu Y.. (2013). Targeting the shift from M1 to M2 macrophages in experimental autoimmune encephalomyelitis mice treated with fasudil. PLoS ONE 8:e54841. 10.1371/journal.pone.0054841 23418431 PMC3572131

[B128] Liu G. Friggeri A. Yang Y. Park Y.-J. Tsuruta Y. Abraham E. (2009). miR-147, a microRNA that is induced upon Toll-like receptor stimulation, regulates murine macrophage inflammatory responses. Proc. Natl. Acad. Sci. U.S.A. 106, 15819–15824. 10.1073/pnas.0901216106 19721002 PMC2747202

[B129] Liu G. H. Qu J. Shen X. (2008). NF-κB/p65 antagonizes Nrf2-ARE pathway by depriving CBP from Nrf2 and facilitating recruitment of HDAC3 to MafK. Biochim. Biophys. Acta Mol. Cell Res. 1783, 713–727. 10.1016/j.bbamcr.2008.01.002 18241676

[B130] Liu H. Y. Chen C. Y. Hsueh Y. P. (2014). Innate immune responses regulate morphogenesis and degeneration: roles of Toll-like receptors and Sarm1 in neurons. Neurosci. Bull. 30, 645–654. 10.1007/s12264-014-1445-5 24993772 PMC5562625

[B131] Liu J. Chen Q. Jian Z. Xiong X. Shao L. Jin T.. (2016). Daphnetin protects against cerebral ischemia/reperfusion injury in mice via inhibition of TLR4/NF-κB signaling pathway. Biomed Res. Int. 2016:2816056. 10.1155/2016/2816056 28119924 PMC5227117

[B132] Liu Q. Li J. Jubair S. Wang D. Luo Y. Fan D.. (2014). Sparstolonin B attenuates hypoxia-induced apoptosis, necrosis and inflammation in cultured rat left ventricular tissue slices. Cardiovasc. Drugs Ther. 28, 433–439. 10.1007/s10557-014-6545-6 25117676 PMC4164598

[B133] Liu X. Gong J. Xu B. (2015). miR-143 down-regulates TLR2 expression in hepatoma cells and inhibits hepatoma cell proliferation and invasion. Int. J. Clin. Exp. Pathol. 8, 12738–12747. 26722463 PMC4680408

[B134] Liu Z. Y. Liu J. Zhao K. L. Wang L. K. Shi Q. Zuo T. (2014). Protective effects of daphnetin on sodium taurocholate-induced severe acute pancreatitis in rats. Mol. Med. Rep. 9, 1709–1714. 10.3892/mmr.2014.1995 24584301

[B135] Ma C. Li Y. Li M. Deng G. Wu X. Zeng J.. (2014). MicroRNA-124 negatively regulates TLR signaling in alveolar macrophages in response to mycobacterial infection. Mol. Immunol. 62, 150–158. 10.1016/j.molimm.2014.06.014 24995397

[B136] Majerova P. Zilkova M. Kazmerova Z. Kovac A. Paholikova K. Kovacech B.. (2014). Microglia display modest phagocytic capacity for extracellular tau oligomers. J. Neuroinflammation 11:161. 10.1186/s12974-014-0161-z 25217135 PMC4172893

[B137] Mao X. Ou M. T. Karuppagounder S. S. Kam T.-I. Yin X. Xiong Y.. (2016). Pathological α-synuclein transmission initiated by binding lymphocyte-activation gene 3. Science 353, aah3374–aah3374. 10.1126/science.aah3374 27708076 PMC5510615

[B138] Matsushima N. Tanaka T. Enkhbayar P. Mikami T. Taga M. Yamada K.. (2007). Comparative sequence analysis of leucine-rich repeats (LRRs) within vertebrate toll-like receptors. BMC Genomics 8:124. 10.1186/1471-2164-8-124 17517123 PMC1899181

[B139] Matteucci C. Minutolo A. Marino-Merlo F. Grelli S. Frezza C. Mastino A.. (2015). Characterization of the enhanced apoptotic response to azidothymidine by pharmacological inhibition of NF-kB. Life Sci. 127, 90–97. 10.1016/j.lfs.2015.01.038 25744407

[B140] McCoy M. K. Martinez T. N. Ruhn K. A. Szymkowski D. E. Smith C. G. Botterman B. R.. (2006). Blocking soluble tumor necrosis factor signaling with dominant-negative tumor necrosis factor inhibitor attenuates loss of dopaminergic neurons in models of Parkinson's disease. J. Neurosci. 26, 9365–9375. 10.1523/JNEUROSCI.1504-06.2006 16971520 PMC3707118

[B141] McKeith I. G. Galasko D. Kosaka K. Perry E. K. Dickson D. W. Hansen L. A.. (1996). Consensus guidelines for the clinical and pathologic diagnosis of dementia with Lewy bodies (DLB): report of the consortium on DLB international workshop. Neurology 47, 1113–1124. 10.1212/WNL.47.5.1113 8909416

[B142] Meisgen F. Xu Landén N. Wang A. Réthi B. Bouez C. Zuccolo M.. (2014). MiR-146a negatively regulates TLR2-induced inflammatory responses in keratinocytes. J. Invest. Dermatol. 134, 1931–1940. 10.1038/jid.2014.89 24670381

[B143] Meng Q. Cai C. Sun T. Wang Q. Xie W. Wang R.. (2015). Reversible ubiquitination shapes NLRC5 function and modulates NF-κB activation switch. J. Cell Biol. 211, 1025–1040. 10.1083/jcb.201505091 26620909 PMC4674279

[B144] Min Y. Wi S. M. Kang J.-A. Yang T. Park C.-S. Park S.-G.. (2016). Cereblon negatively regulates TLR4 signaling through the attenuation of ubiquitination of TRAF6. Cell Death Dis. 7:e2313. 10.1038/cddis.2016.226 27468689 PMC4973362

[B145] Mirshafiey A. Hosseini S. Afraei S. Rastkari N. Zavareh F. Azizi G. (2016). Anti-aging property of G2013 molecule as a novel immuno- suppressive agent on enzymatic and non-enzymatic oxidative stress determinants in rat Model. Curr Drug Discov Technol. 13, 25–33. 10.2174/1570163813666160224123851 26906909

[B146] Mistry P. Laird M. H. W. Schwarz R. S. Greene S. Dyson T. Snyder G. A.. (2015). Inhibition of TLR2 signaling by small molecule inhibitors targeting a pocket within the TLR2 TIR domain. Proc. Natl. Acad. Sci. U.S.A. 112, 5455–5460. 10.1073/pnas.1422576112 25870276 PMC4418912

[B147] Morganti J. M. Riparip L. K. Rosi S. (2016). Call off the dog(ma): M1/M2 polarization is concurrent following traumatic brain injury. PLoS ONE 11:e0148001. 10.1371/journal.pone.0148001 26808663 PMC4726527

[B148] Morr M. Takeuchi O. Akira S. Simon M. M. Mühlradt P. F. (2002). Differential recognition of structural details of bacterial lipopeptides by toll-like receptors. Eur. J. Immunol. 32, 3337–47. 10.1002/1521-4141(2002012)32:12<3337::AID-IMMU3337>3.0.CO;2-I 12432564

[B149] Nahid M. A. Pauley K. M. Satoh M. Chan E. K. L. (2009). miR-146a is critical for endotoxin-induced tolerance: implication in innate immunity. J. Biol. Chem. 284, 34590–34599. 10.1074/jbc.M109.056317 19840932 PMC2787321

[B150] Nahid M. A. Satoh M. Chan E. K. L. (2011). Mechanistic role of microRNA-146a in endotoxin-induced differential cross-regulation of TLR signaling. J. Immunol. 186, 1723–1734. 10.4049/jimmunol.1002311 21178010 PMC3608687

[B151] Nahid M. A. Yao B. Dominguez-Gutierrez P. R. Kesavalu L. Satoh M. Chan E. K. L. (2013). Regulation of TLR2-mediated tolerance and cross-tolerance through IRAK4 modulation by miR-132 and miR-212. J. Immunol. 190, 1250–1263. 10.4049/jimmunol.1103060 23264652 PMC3552145

[B152] Nakagawa R. Naka T. Tsutsui H. Fujimoto M. Kimura A. Abe T.. (2002). SOCS-1 participates in negative regulation of LPS responses. Immunity 17, 677–687. 10.1016/S1074-7613(02)00449-1 12433373

[B153] Nazeri S. Khadem Azarian S. Fattahi M. J. Sedaghat R. Tofighi Zavareh F. Aghazadeh Z.. (2017). Preclinical and pharmacotoxicology evaluation of α-l-guluronic acid (G2013) as a non-steroidal anti-inflammatory drug with immunomodulatory property. Immunopharmacol. Immunotoxicol. 39, 59–65. 10.1080/08923973.2017.1282512 28145788

[B154] Neher J. J. Neniskyte U. Zhao J.-W. Bal-Price A. Tolkovsky A. M. Brown G. C. (2011). Inhibition of microglial phagocytosis is sufficient to prevent inflammatory neuronal death. J. Immunol. 186, 4973–4983. 10.4049/jimmunol.1003600 21402900

[B155] Nicotra L. Loram L. C. Watkins L. R. Hutchinson M. R. (2012). Toll-like receptors in chronic pain. Exp. Neurol. 234, 316–329. 10.1016/j.expneurol.2011.09.038 22001158 PMC3303935

[B156] Nimmerjahn A. Kirchhoff F. Helmchen F. (2005). Resting microglial cells are highly dynamic surveillants of brain parenchyma *in vivo*. Science 308, 1314–1318. 10.1126/science.1110647 15831717

[B157] Nishiguchi M. Matsumoto M. Takao T. Hoshino M. Shimonishi Y. Tsuji S.. (2001). Mycoplasma fermentans lipoprotein M161Ag-induced cell activation is mediated by toll-like receptor 2: role of n-terminal hydrophobic portion in its multiple functions. J. Immunol. 166, 2610–2616. 10.4049/jimmunol.166.4.2610 11160323

[B158] O'Connell R. M. Taganov K. D. Boldin M. P. Cheng G. Baltimore D. (2007). MicroRNA-155 is induced during the macrophage inflammatory response. Proc. Natl. Acad. Sci. U.S.A. 104, 1604–1609. 10.1073/pnas.0610731104 17242365 PMC1780072

[B159] O'Neill L. A. Sheedy F. J. McCoy C. E. (2011). MicroRNAs: the fine-tuners of Toll-like receptor signalling. Nat. Rev. Immunol. 11, 163–175. 10.1038/nri2957 21331081

[B160] Okusawa T. Fujita M. Nakamura J. Into T. Yasuda M. Yoshimura A.. (2004). Relationship between structures and biological activities of mycoplasmal diacylated lipopeptides and their recognition by toll-like receptors 2 and 6. Infect. Immun. 72, 1657–1665. 10.1128/IAI.72.3.1657-1665.2004 14977973 PMC355991

[B161] Olajide O. J. Asogwa N. T. Moses B. O. Oyegbola C. B. (2017). Multidirectional inhibition of cortico-hippocampal neurodegeneration by kolaviron treatment in rats. Metab. Brain Dis. 32, 1147–1161 10.1007/s11011-017-0012-6 28405779

[B162] Onasanwo S. A. Velagapudi R. El-Bakoush A. Olajide O. A. (2016). Inhibition of neuroinflammation in BV2 microglia by the biflavonoid kolaviron is dependent on the Nrf2/ARE antioxidant protective mechanism. Mol. Cell. Biochem. 414, 23–36. 10.1007/s11010-016-2655-8 26838169

[B163] Oshiumi H. Matsumoto M. Funami K. Akazawa T. Seya T. (2003). TICAM-1, an adaptor molecule that participates in Toll-like receptor 3-mediated interferon-β induction. Nat. Immunol. 4, 161–167. 10.1038/ni886 12539043

[B164] Ouchi Y. Yagi S. Yokokura M. Sakamoto M. (2009). Neuroinflammation in the living brain of Parkinson's disease. Park. Relat. Disord. 15, S200–S204. 10.1016/S1353-8020(09)70814-4 20082990

[B165] Ouchi Y. Yoshikawa E. Sekine Y. Futatsubashi M. Kanno T. Ogusu T.. (2005). Microglial activation and dopamine terminal loss in early Parkinson's disease. Ann. Neurol. 57, 168–175. 10.1002/ana.20338 15668962

[B166] Oyagbemi A. A. Bester D. Esterhuyse J. Farombi E. O. (2017). Kolaviron, a biflavonoid of Garcinia kola seed mitigates ischemic/reperfusion injury by modulation of pro-survival and apoptotic signaling pathways. J. Intercult. Ethnopharmacol. 6, 42–49. 10.5455/jice.20160923100223 28163959 PMC5289087

[B167] Ozinsky A. Underhill D. M. Fontenot J. D. Hajjar A. M. Smith K. D. Wilson C. B.. (2000). The repertoire for pattern recognition of pathogens by the innate immune system is defined by cooperation between toll-like receptors. Proc. Natl. Acad. Sci. U.S.A. 97, 13766–13771. 10.1073/pnas.250476497 11095740 PMC17650

[B168] Park S. Y. Bae Y. S. Ko M. J. Lee S. J. Choi Y. W. (2014). Comparison of anti-inflammatory potential of four different dibenzocyclooctadiene lignans in microglia; action via activation of PKA and Nrf-2 signaling and inhibition of MAPK/STAT/NF-κB pathways. Mol. Nutr. Food Res. 58, 738–748. 10.1002/mnfr.201300445 24214854

[B169] Park S. Y. Kim Y. H. Park G. (2015). Cucurbitacins attenuate microglial activation and protect from neuroinflammatory injury through Nrf2/ARE activation and STAT/NF-κB inhibition. Neurosci. Lett. 609, 129–136. 10.1016/j.neulet.2015.10.022 26472707

[B170] Pauls E. Shpiro N. Peggie M. Young E. R. Sorcek R. J. Tan L.. (2012). Essential role for IKKβ in production of type 1 interferons by plasmacytoid dendritic cells. J. Biol. Chem. 287, 19216–19228. 10.1074/jbc.M112.345405 22511786 PMC3365954

[B171] Perrin R. J. Woods W. S. Clayton D. F. George J. M. (2001). Exposure to long chain polyunsaturated fatty acids triggers rapid multimerization of synucleins. J. Biol. Chem. 276, 41958–41962. 10.1074/jbc.M105022200 11553616

[B172] Poltorak A. He X. Smirnova I. Liu M. Y. Van Huffel C. Du X.. (1998). Defective LPS signaling in C3H/HeJ and C57BL/10ScCr mice: mutations in Tlr4 gene. Science 282, 2085–2088. 10.1126/science.282.5396.2085 9851930

[B173] Polymeropoulos M. H. Higgins J. J. Golbe L. I. Johnson W. G. Ide S. E. Di Iorio G.. (1996). Mapping of a gene for Parkinson's disease to chromosome 4q21-q23. Science 274, 1197–1199. 8895469 10.1126/science.274.5290.1197

[B174] Polymeropoulos M. H. Lavedan C. Leroy E. Ide S. E. Dehejia A. Dutra A.. (1997). Mutation in the α-synuclein gene identified in families with Parkinson's disease. Science 276, 2045–2047. 9197268 10.1126/science.276.5321.2045

[B175] Pourgholi F. Hajivalili M. Razavi R. Esmaeili S. Baradaran B. Movasaghpour A. A.. (2017). The role of M2000 as an anti-inflammatory agent in toll-like receptor 2/microRNA-155 pathway. Avicenna J. Med. Biotechnol. 9, 8–12. 28090274 PMC5219823

[B176] Qiu S. Feng Y. LeSage G. Zhang Y. Stuart C. He L.. (2015). Chronic morphine-induced microRNA-124 promotes microglial immunosuppression by modulating P65 and TRAF6. J. Immunol. 194, 1021–1030. 10.4049/jimmunol.1400106 25539811 PMC4297711

[B177] Quinn E. M. Wang J. H. O'Callaghan G. Redmond H. P. (2013). MicroRNA-146a is upregulated by and negatively regulates TLR2 signaling. PLoS ONE 8:e62232. 10.1371/journal.pone.0062232 23638011 PMC3639252

[B178] Ransohoff R. M. (2016). A polarizing question: do M1 and M2 microglia exist? Nat. Neurosci. 19, 987–991. 10.1038/nn.4338 27459405

[B179] Reed-Geaghan E. G. Savage J. C. Hise A. G. Landreth G. E. (2009). CD14 and toll-like receptors 2 and 4 are required for fibrillar Aβ-stimulated microglial activation. J. Neurosci. 29, 11982–11992. 10.1523/JNEUROSCI.3158-09.2009 19776284 PMC2778845

[B180] Rice T. W. Wheeler A. P. Bernard G. R. Vincent J.-L. Angus D. C. Aikawa N.. (2010). A randomized, double-blind, placebo-controlled trial of TAK-242 for the treatment of severe sepsis^*^. Crit. Care Med. 38, 1685–1694. 10.1097/CCM.0b013e3181e7c5c9 20562702

[B181] Roodveldt C. Labrador-Garrido A. Gonzalez-Rey E. Lachaud C. C. Guilliams T. Fernandez-Montesinos R.. (2013). Preconditioning of microglia by α-synuclein strongly affects the response induced by toll-like receptor (TLR) stimulation. PLoS ONE 8:e79160. 10.1371/journal.pone.0079160 24236103 PMC3827304

[B182] Rossignol D. P. Lynn M. (2002). Antagonism of *in vivo* and *ex vivo* response to endotoxin by E5564, a synthetic lipid A analogue. J. Endotoxin Res. 8, 483–488. 10.1177/09680519020080060601 12697095

[B183] Saba R. Gushue S. Huzarewich R. L. C. H. Manguiat K. Medina S. Robertson C.. (2012). MicroRNA 146a (miR-146a) is over-expressed during prion disease and modulates the innate immune response and the microglial activation state. PLoS ONE 7:e30832. 10.1371/journal.pone.0030832 22363497 PMC3281888

[B184] Sandor F. Latz E. Re F. Mandell L. Repik G. Golenbock D. T. (2003). Importance of extra- and intracellular domains of TLR1 and TLR2 in NFκB signaling. J. Cell Biol. 162, 1099–1110. 10.1083/jcb.200304093 12975352 PMC2172862

[B185] Santra M. Zhang Z. G. Yang J. Santra S. Santra S. Chopp M.. (2014). Thymosin β4 up-regulation of MicroRNA-146a promotes oligodendrocyte differentiation and suppression of the toll-like proinflammatory pathway. J. Biol. Chem. 289, 19508–19518. 10.1074/jbc.M113.529966 24828499 PMC4094061

[B186] Shah J. A. Vary J. C. Chau T. T. H. Bang N. D. Yen N. T. B. Farrar J. J.. (2012). Human TOLLIP regulates TLR2 and TLR4 signaling and its polymorphisms are associated with susceptibility to tuberculosis. J. Immunol. 189, 1737–1746. 10.4049/jimmunol.1103541 22778396 PMC3428135

[B187] Sharifi L. Mohsenzadegan M. Aghamohammadi A. Rezaei N. Tofighi Zavareh F. Bokaie S.. (2017). Immunomodulatory effect of G2013 (α-L-Guluronic acid) on the TLR2 and TLR4 in human mononuclear cells. Curr. Drug Discov. Technol. [Epub ahead of print]. 10.2174/1570163814666170605111331 28578651

[B188] Sharon R. Bar-Joseph I. Frosch M. P. Walsh D. M. Hamilton J. A. Selkoe D. J. (2003). The formation of highly soluble oligomers of α-synuclein is regulated by fatty acids and enhanced in Parkinson's disease. Neuron 37, 583–595. 10.1016/S0896-6273(03)00024-2 12597857

[B189] Shechter R. London A. Kuperman Y. Ronen A. Rolls A. Chen A.. (2013). Hypothalamic neuronal toll-like receptor 2 protects against age-induced obesity. Sci. Rep. 3:1254. 10.1038/srep01254 23409245 PMC3570778

[B190] Shtilerman M. D. Ding T. T. Lansbury P. T. (2002). Molecular crowding accelerates fibrillization of α-synuclein: could an increase in the cytoplasmic protein concentration induce Parkinson's disease? Biochemistry 41, 3855–3860. 10.1021/bi0120906 11900526

[B191] Singleton A. B. Farrer M. Johnson J. Singleton A. Hague S. Kachergus J.. (2003). α-Synuclein locus triplication causes Parkinson's disease. Science 302:841. 10.1126/science.1090278 14593171

[B192] Song F. Zeng K.-W. Liao L. Yu Q. Tu P. Wang X. (2016). Schizandrin A inhibits microglia-mediated neuroninflammation through inhibiting TRAF6-NF-κB and Jak2-Stat3 signaling pathways. PLoS ONE 11:e0149991. 10.1371/journal.pone.0149991 26919063 PMC4768966

[B193] Spillantini M. G. Crowther R. A. Jakes R. Hasegawa M. Goedert M. (1998). α-Synuclein in filamentous inclusions of Lewy bodies from Parkinson's disease and dementia with lewy bodies. Proc. Natl. Acad. Sci. U.S.A. 95, 6469–6473. 9600990 10.1073/pnas.95.11.6469 PMC27806

[B194] Spillantini M. G. Schmidt M. L. Lee V. M. Trojanowski J. Q. Jakes R. Goedert M. (1997). α-synuclein in Lewy bodies. Nature 388, 839–840. 10.1038/42166 9278044

[B195] Stefanova N. Fellner L. Reindl M. Masliah E. Poewe W. Wenning G. K. (2011). Toll-like receptor 4 promotes α-synuclein clearance and survival of nigral dopaminergic neurons. Am. J. Pathol. 179, 954–963. 10.1016/j.ajpath.2011.04.013 21801874 PMC3157205

[B196] Strassheim D. Kim J.-Y. Park J.-S. Mitra S. Abraham E. (2005). Involvement of SHIP in TLR2-induced neutrophil activation and acute lung injury. J. Immunol. 174, 8064–8071. 10.4049/jimmunol.174.12.8064 15944314

[B197] Su Q. Grabowski M. Weindl G. (2017). Recognition of Propionibacterium acnes by human TLR2 heterodimers. Int. J. Med. Microbiol. 307, 108–112. 10.1016/j.ijmm.2016.12.002 28024924

[B198] Su X. Maguire-Zeiss K. A. Giuliano R. Prifti L. Venkatesh K. Federoff H. J. (2008). Synuclein activates microglia in a model of Parkinson's disease. Neurobiol. Aging 29, 1690–1701. 10.1016/j.neurobiolaging.2007.04.006 17537546 PMC2621109

[B199] Syed M. M. Phulwani N. K. Kielian T. (2007). Tumor necrosis factor-α (TNF-α) regulates Toll-like receptor 2 (TLR2) expression in microglia. J. Neurochem. 103, 1461–1471. 10.1111/j.1471-4159.2007.04838.x 17961202 PMC2423670

[B200] Symons A. Beinke S. Ley S. C. (2006). MAP kinase kinase kinases and innate immunity. Trends Immunol. 27, 40–48. 10.1016/j.it.2005.11.007 16356769

[B201] Taganov K. D. Boldin M. P. Chang K.-J. Baltimore D. (2006). NF-κB-dependent induction of microRNA miR-146, an inhibitor targeted to signaling proteins of innate immune responses. Proc. Natl. Acad. Sci. U.S.A. 103, 12481–12486. 10.1073/pnas.0605298103 16885212 PMC1567904

[B202] Takeuchi H. Jin S. Wang J. Zhang G. Kawanokuchi J. Kuno R.. (2006). Tumor necrosis factor-α induces neurotoxicity via glutamate release from hemichannels of activated microglia in an autocrine manner. J. Biol. Chem. 281, 21362–21368. 10.1074/jbc.M600504200 16720574

[B203] Takeuchi O. Hoshino K. Akira S. (2000). Cutting Edge: TLR2-deficient and MyD88-deficient mice are highly susceptible to *Staphylococcus aureus* infection. J. Immunol. 165, 5392–5396. 10.4049/jimmunol.165.10.5392 11067888

[B204] Takeuchi O. Kawai T. Mühlradt P. F. Morr M. Radolf J. D. Zychlinsky A.. (2001). Discrimination of bacterial lipoproteins by Toll-like receptor 6. Int. Immunol. 13, 933–940. 10.1093/intimm/13.7.933 11431423

[B205] Takeuchi O. Sato S. Horiuchi T. Hoshino K. Takeda K. Dong Z.. (2002). Cutting edge: role of Toll-like receptor 1 in mediating immune response to microbial lipoproteins. J. Immunol. 169, 10–14. 10.4049/jimmunol.169.1.10 12077222

[B206] Tanga F. Y. Nutile-McMenemy N. DeLeo J. A. (2005). The CNS role of Toll-like receptor 4 in innate neuroimmunity and painful neuropathy. Proc. Natl. Acad. Sci. U.S.A. 102, 5856–5861. 10.1073/pnas.0501634102 15809417 PMC556308

[B207] Thounaojam M. C. Kundu K. Kaushik D. K. Swaroop S. Mahadevan A. Shankar S. K.. (2014). MicroRNA 155 regulates Japanese encephalitis virus-induced inflammatory response by targeting Src homology 2-containing inositol phosphatase 1. J. Virol. 88, 4798–4810. 10.1128/JVI.02979-13 24522920 PMC3993824

[B208] Tsigelny I. F. Sharikov Y. Miller M. A. Masliah E. (2008). Mechanism of α-synuclein oligomerization and membrane interaction: theoretical approach to unstructured proteins studies. Nanomedicine Nanotechnology, Biol. Med. 4, 350–357. 10.1016/j.nano.2008.05.005 18640077 PMC2627560

[B209] Tu L. Li S. Fu Y. Yao R. Zhang Z. Yang S.. (2012). The therapeutic effects of daphnetin in collagen-induced arthritis involve its regulation of Th17 cells. Int. Immunopharmacol. 13, 417–423. 10.1016/j.intimp.2012.04.001 22554834

[B210] Underhill D. M. Ozinsky A. Hajjar A. M. Stevens A. Wilson C. B. Bassetti M.. (1999a). The Toll-like receptor 2 is recruited to macrophage phagosomes and discriminates between pathogens. Nature 401, 811–815. 10548109 10.1038/44605

[B211] Underhill D. M. Ozinsky A. Smith K. D. Aderem A. (1999b). Toll-like receptor-2 mediates mycobacteria-induced proinflammatory signaling in macrophages. Proc. Natl. Acad. Sci. U.S.A. 96, 14459–14463. 10588727 10.1073/pnas.96.25.14459 PMC24458

[B212] Uversky V. N. (2007). Neuropathology, biochemistry, and biophysics of α-synuclein aggregation. J. Neurochem. 103, 17–37. 10.1111/j.1471-4159.2007.04764.x 17623039

[B213] Uversky V. N. (2011a). Intrinsically disordered proteins from A to Z. Int. J. Biochem. Cell Biol. 43, 1090–1103. 10.1016/j.biocel.2011.04.001 21501695

[B214] Uversky V. N. (2011b). Intrinsically disordered proteins may escape unwanted interactions via functional misfolding. Biochim. Biophys. Acta Proteins Proteomics 1814, 693–712. 10.1016/j.bbapap.2011.03.010 21440685

[B215] Vezzani A. Maroso M. Balosso S. Sanchez M. A. Bartfai T. (2011). IL-1 receptor/Toll-like receptor signaling in infection, inflammation, stress and neurodegeneration couples hyperexcitability and seizures. Brain. Behav. Immun. 25, 1281–1289. 10.1016/j.bbi.2011.03.018 21473909

[B216] Vo N. Klein M. E. Varlamova O. Keller D. M. Yamamoto T. Goodman R. H.. (2005). A cAMP-response element binding protein-induced microRNA regulates neuronal morphogenesis. Proc. Natl. Acad. Sci. U.S.A. 102, 16426–16431. 10.1073/pnas.0508448102 16260724 PMC1283476

[B217] Volles M. J. Lansbury P. T. (2002). Vesicle permeabilization by protofibrillar α-synuclein is sensitive to Parkinson's disease-linked mutations and occurs by a pore-like mechanism. Biochemistry 41, 4595–4602. 10.1021/bi0121353 11926821

[B218] Volles M. J. Lee S.-J. Rochet J.-C. Shtilerman M. D. Ding T. T. Kessler J. C.. (2001). Vesicle permeabilization by protofibrillar α-synuclein: implications for the pathogenesis and treatment of Parkinson's disease. Biochemistry 40, 7812–7819. 10.1021/bi0102398 11425308

[B219] Wang L. H. Johnson E. M. Shoulson I. Lang A. E. Bozyczko-Coyne D. (2008). Mixed lineage kinase inhibitor CEP-1347 fails to delay disability in early Parkinson disease. Neurology 71, 462–463. 10.1212/01.wnl.0000324506.93877.5e 18678833

[B220] Wang X. Wang C. Wang J. Zhao S. Zhang K. Wang J.. (2014). Pseudoginsenoside-F11 (PF11) exerts anti-neuroinflammatory effects on LPS-activated microglial cells by inhibiting TLR4-mediated TAK1/IKK/NF-κB, MAPKs and Akt signaling pathways. Neuropharmacology 79, 642–656. 10.1016/j.neuropharm.2014.01.022 24467851

[B221] Wayman G. A. Davare M. Ando H. Fortin D. Varlamova O. Cheng H.-Y. M.. (2008). An activity-regulated microRNA controls dendritic plasticity by down-regulating p250GAP. Proc. Natl. Acad. Sci. U.S.A. 105, 9093–9098. 10.1073/pnas.0803072105 18577589 PMC2449370

[B222] Wei Z. Chigurupati S. Arumugam T. V. Jo D. G. Li H. Chan S. L. (2011). Notch activation enhances the microglia-mediated inflammatory response associated with focal cerebral ischemia. Stroke 42, 2589–2594. 10.1161/STROKEAHA.111.614834 21737799

[B223] West A. P. Koblansky A. A. Ghosh S. (2006). Recognition and signaling by toll-like receptors. Annu. Rev. Cell Dev. Biol. 22, 409–437. 10.1146/annurev.cellbio.21.122303.115827 16822173

[B224] Wightman B. Bürglin T. R. Gatto J. Arasu P. Ruvkun G. (1991). Negative regulatory sequences in the lin-14 3′-untranslated region are necessary to generate a temporal switch during Caenorhabditis elegans development. Genes Dev. 5, 1813–1824. 10.1101/gad.5.10.1813 1916264

[B225] Wightman B. Ha I. Ruvkun G. (1993). Posttranscriptional regulation of the heterochronic gene lin-14 by lin-4 mediates temporal pattern formation in *C. elegans*. Cell 75, 855–862. 10.1016/0092-8674(93)90530-4 8252622

[B226] Xiao C. Rajewsky K. (2009). MicroRNA control in the immune system: basic principles. Cell 136, 26–36. 10.1016/j.cell.2008.12.027 19135886

[B227] Xu J. Kao S.-Y. Lee F. J. S. Song W. Jin L.-W. Yankner B. A. (2002). Dopamine-dependent neurotoxicity of α-synuclein: a mechanism for selective neurodegeneration in Parkinson disease. Nat. Med. 8, 600–606. 10.1038/nm0602-600 12042811

[B228] Xu Y. Tao X. Shen B. Horng T. Medzhitov R. Manley J. L.. (2000). Structural basis for signal transduction by the Toll/interleukin-1 receptor domains. Nature 408, 111–115. 10.1038/35040600 11081518

[B229] Yamamoto M. Sato S. Hemmi H. Sanjo H. Uematsu S. Kaisho T.. (2002a). Essential role for TIRAP in activation of the signalling cascade shared by TLR2 and TLR4. Nature 420, 324–329. 10.1038/nature01182 12447441

[B230] Yamamoto M. Sato S. Mori K. Hoshino K. Takeuchi O. Takeda K.. (2002b). Cutting edge: a novel toll/IL-1 receptor domain-containing adapter that preferentially activates the IFN-β promoter in the toll-like receptor signaling. J. Immunol. 169, 6668–6672. 10.4049/jimmunol.169.12.6668 12471095

[B231] Yang J. Amiri K. I. Burke J. R. Schmid J. A. Richmond A. (2006). BMS-345541 targets inhibitor of κB kinase and induces apoptosis in melanoma: involvement of nuclear factor κB and mitochondria pathways. Clin. Cancer Res. 12, 950–960. 10.1158/1078-0432.CCR-05-1220 16467110 PMC2668250

[B232] Yang W.-L. Wang J. Chan C.-H. Lee S.-W. Campos A. D. Lamothe B.. (2009). The E3 ligase TRAF6 regulates Akt ubiquitination and activation. Science 325, 1134–1138. 10.1126/science.1175065 19713527 PMC3008763

[B233] Yao R. Fu Y. Li S. Tu L. Zeng X. Kuang N. (2011). Regulatory effect of daphnetin, a coumarin extracted from Daphne odora, on the balance of Treg and Th17 in collagen-induced arthritis. Eur. J. Pharmacol. 670, 286–294. 10.1016/j.ejphar.2011.08.019 21914445

[B234] Yin Q. McBride J. Fewell C. Lacey M. Wang X. Lin Z.. (2008). MicroRNA-155 is an epstein-barr virus-induced gene that modulates epstein-barr virus-regulated gene expression pathways. J. Virol. 82, 5295–5306. 10.1128/JVI.02380-07 18367535 PMC2395216

[B235] Yu L. Wang L. Chen S. (2010). Endogenous toll-like receptor ligands and their biological significance. J. Cell. Mol. Med. 14, 2592–2603. 10.1111/j.1582-4934.2010.01127.x 20629986 PMC4373479

[B236] Yu W. Lu Z. Zhang H. Kang Y. Mao Y. Wang H.. (2014). Anti-inflammatory and protective properties of daphnetin in endotoxin-induced lung injury. J. Agric. Food Chem. 62, 12315–12325. 10.1021/jf503667v 25419854

[B237] Zeng K. W. Yu Q. Song F. J. Liao L. X. Zhao M. B. Dong X.. (2015). Deoxysappanone B, a homoisoflavone from the Chinese medicinal plant *Caesalpinia sappan* L., protects neurons from microglia-mediated inflammatory injuries via inhibition of IκB kinase (IKK)-NF-κB and p38/ERK MAPK pathways. Eur. J. Pharmacol. 748, 18–29. 10.1016/j.ejphar.2014.12.013 25530267

[B238] Zeng K. W. Zhang T. Fu H. Liu G. X. Wang X. M. (2012). Schisandrin B exerts anti-neuroinflammatory activity by inhibiting the Toll-like receptor 4-dependent MyD88/IKK/NF-κB signaling pathway in lipopolysaccharide-induced microglia. Eur. J. Pharmacol. 692, 29–37. 10.1016/j.ejphar.2012.05.030 22698579

[B239] Zhang G. Ghosh S. (2002). Negative regulation of toll-like receptor-mediated signaling by Tollip. J. Biol. Chem. 277, 7059–7065. 10.1074/jbc.M109537200 11751856

[B240] Zhang W. W. Wang T. Pei Z. Miller D. S. Wu X. Block M. L.. (2005). Aggregated α-synuclein activates microglia: a process leading to disease progression in Parkinson's disease. FASEB J. 19, 533–542. 10.1096/fj.04-2751com 15791003

[B241] Zhang Y. Zhang H. E. Liu Z. (2016). MicroRNA-147 suppresses proliferation, invasion and migration through the AKT/mTOR signaling pathway in breast cancer. Oncol. Lett. 11, 405–410. 10.3892/ol.2015.3842 26870225 PMC4727187

[B242] Zhao L. Zabel M. K. Wang X. Ma W. Shah P. Fariss R. N.. (2015). Microglial phagocytosis of living photoreceptors contributes to inherited retinal degeneration. EMBO Mol. Med. 7, 1179–1197. 10.15252/emmm.201505298 26139610 PMC4568951

[B243] Zhao Y. F. Zhang Q. Xi J. Y. Li Y. H. Ma C. G. Xiao B. G. (2015). Multitarget intervention of Fasudil in the neuroprotection of dopaminergic neurons in MPTP-mouse model of Parkinson's disease. J. Neurol. Sci. 353, 28–37. 10.1016/j.jns.2015.03.022 25908255

[B244] Zhou T. Huang Y.-X. Song J.-W. Ma Q.-M. (2015). Thymosin β4 inhibits microglia activation through microRNA 146a in neonatal rats following hypoxia injury. Neuroreport 26, 1032–1038. 10.1097/WNR.0000000000000463 26457369

